# Efficacy and cost-effectiveness of a community-based smoke-free-home intervention with or without indoor-air-quality feedback in Bangladesh (MCLASS II): a three-arm, cluster-randomised, controlled trial

**DOI:** 10.1016/S2214-109X(21)00040-1

**Published:** 2021-04-15

**Authors:** Noreen Dadirai Mdege, Caroline Fairhurst, Han-I Wang, Tarana Ferdous, Anna-Marie Marshall, Catherine Hewitt, Rumana Huque, Cath Jackson, Ian Kellar, Steve Parrott, Sean Semple, Aziz Sheikh, Qi Wu, Zunayed Al Azdi, Kamran Siddiqi, MCLASS II trial team

**Affiliations:** a Department of Health Sciences, University of York, York, UK; b York Trials Unit, University of York, York, UK; c Faculty of Sciences, and Hull York Medical School, University of York, York, UK; d ARK Foundation, Dhaka, Bangladesh; e Department of Economics, Dhaka University, Dhaka, Bangladesh; f Valid Research, Leeds, UK; g School of Psychology, Faculty of Medicine and Health, University of Leeds, Leeds, UK; h Institute for Social Marketing and Health, University of Stirling, Stirling, UK; i Usher Institute, University of Edinburgh, Edinburgh, UK

## Abstract

**Background:**

Exposure to second-hand smoke from tobacco is a major contributor to global morbidity and mortality. We aimed to evaluate the efficacy and cost-effectiveness of a community-based smoke-free-home intervention, with or without indoor-air-quality feedback, in reducing second-hand-smoke exposure in homes in Bangladesh.

**Methods:**

We did a three-arm, cluster-randomised, controlled trial in Dhaka, Bangladesh, and randomly assigned (1:1:1) mosques and consenting households from their congregations to a smoke-free-home intervention plus indoor-air-quality feedback, smoke-free-home intervention only, or usual services. Households were eligible if they had at least one resident attending one of the participating mosques, at least one adult resident (age 18 years or older) who smoked cigarettes or other forms of smoked tobacco (eg, bidi, waterpipe) regularly (on at least 25 days per month), and at least one non-smoking resident of any age. The smoke-free-home intervention consisted of weekly health messages delivered within an Islamic discourse by religious leaders at mosques over 12 weeks. Indoor-air-quality feedback comprised providing households with feedback on their indoor air quality measured over 24 h. Households in the usual services group received no intervention. Masking of participants and mosque leaders was not possible. The primary outcome was the 24-h mean household airborne fine particulate matter (<2·5 microns in diameter [PM_2·5_]) concentration (a marker of second-hand smoke) at 12 months after randomisation. Cost-effectiveness was estimated using incremental cost-effectiveness ratios (ICERs). This trial is registered with ISRCTN, 49975452.

**Findings:**

Between April 11 and Aug 2, 2018, we enrolled 1801 households from 45 mosques. 640 households (35·5%) were assigned to the smoke-free-home intervention plus indoor-air-quality feedback group, 560 (31·1%) to the smoke-free-home intervention only group, and 601 (33·4%) to the usual services group. At 12 months, the adjusted mean difference in household mean 24-h PM_2·5_ concentration was −1·0 μg/m^3^ (95% CI −12·8 to 10·9, p=0·88) for the smoke-free-home intervention plus indoor-air-quality feedback group versus the usual services group, 5·0 μg/m^3^ (–7·9 to 18·0, p=0·45) for the smoke-free-home intervention only group versus the usual services group, and −6·0 μg/m^3^ (–18·3 to 6·3, p=0·34) for the smoke-free-home intervention plus indoor-air-quality feedback group versus the smoke-free-home intervention only group. The ICER for the smoke-free-home intervention plus indoor-air-quality feedback versus usual services was US$653 per quality-adjusted life-year (QALY) gained, which was more than the upper limit of the Bangladesh willingness-to-pay threshold of $427 per QALY.

**Interpretation:**

The smoke-free-home intervention, with or without indoor-air-quality feedback, was neither effective nor cost-effective in reducing household second-hand-smoke exposure compared with usual services. These interventions are therefore not recommended for Bangladesh.

**Funding:**

Medical Research Council UK.

**Translation:**

For the Bengali translation of the abstract see Supplementary Materials section.

## Introduction

Approximately 1·2 million people worldwide die from exposure to second-hand tobacco smoke every year.^1^ Around 47% of these deaths occur in women and 28% in children, and most occur in low-income and middle-income countries (LMICs).^2^ About 11 million disability-adjusted life-years are lost due to second-hand-smoke exposure worldwide every year, and children bear approximately 61% of the burden of disease attributable to second-hand smoke.^2^ In Bangladesh, 40·8 million adults (approximately 39%) and 31% of students age 12–16 years (school year groups 7–9) are exposed to second-hand smoke in their homes.^3^, ^4^ A 2015 survey of 12 schools in Dhaka, Bangladesh, found that 95% of children age 9–11 years had saliva cotinine levels consistent with second-hand-smoke exposure.^5^ The mean cotinine value of children living with a smoker was approximately double that of those not living with a smoker.^5^ Thus, homes remain a key source of second-hand-smoke exposure for children in Bangladesh.

**Research in context**

**Evidence before this study**

We reviewed relevant literature identified from four databases: MEDLINE, Embase, CINAHL, and the Cochrane Central Register of Controlled Trials. We searched for randomised controlled trials of community-based interventions to reduce second-hand-smoke exposure from low-income and middle-income countries (LMICs), using the search terms “tobacco smoke pollution”, “passive smoke”, “indoor air pollution”, “environmental tobacco smoke”, and “second-hand smoke”. The studies had to have reported biochemically verified second-hand-smoke exposure (eg, through measuring air quality, air nicotine concentrations, or cotinine concentration in the blood, urine, saliva, or hair) as an outcome. We searched for English language publications from the inception of each database until July, 2017, and did updated searches on Dec 18, 2018. We found six studies that met our eligibility criteria. All six studies evaluated counselling or educational interventions targeted at reducing second-hand-smoke exposure, particularly among children. Three studies evaluated interventions delivered to children within schools. Of these, two studies from China found that the interventions were effective in reducing mean urine cotinine concentration, and the remaining study from Bangladesh (done by our team) did not find any significant difference between groups on saliva cotinine concentrations. A study from China, where an intervention was delivered in people's homes, found that the intervention was effective in reducing mean urine cotinine concentration among children. Another study from Iran identified participants from health centres and showed that a community-based intervention was effective in reducing mean urine cotinine concentrations among children. The sixth study, done in Armenia, did not find any benefits from an intervention in terms of hair cotinine concentrations. Our review concluded that, although there is some evidence of the effectiveness of community-based interventions in reducing second-hand-smoke exposure, this evidence is scarce. Moreover, the potential of religion in promoting behaviours that are protective from second-hand-smoke exposure was yet to be explored. We did not identify any studies evaluating the costs or cost-effectiveness of community-based interventions to reduce second-hand-smoke exposure.

**Added value of this study**

To our knowledge, our trial is the first to investigate the efficacy and cost-effectiveness of community-based interventions, delivered within a faith-based discourse by imams and other religious leaders in mosques, with or without an individual-level indoor-air-quality feedback intervention, for reducing second-hand-smoke exposure within households. We found that the interventions were neither effective nor cost-effective when compared with usual services. However, we showed that it is feasible to do large studies of such interventions within faith-based settings in low-income countries.

**Implications of all the available evidence**

Current evidence on the effectiveness and cost-effectiveness of community-based interventions to reduce second-hand-smoke exposure in LMICs is scarce, and the findings are mixed. Unless future studies provide strong evidence for their effectiveness and cost-effectiveness, such interventions cannot be recommended for use within LMIC settings. There is a need for further studies to explore interventions that have shown promise in high-income countries, such as those that combine smoke-free-home interventions with smoking cessation advice and support for smokers within the home.

88% of the total population of Bangladesh are Muslim.^6^ Religion has an influence on both health-risk behaviours and health,^7^, ^8^, ^9^ and is an important conveyor of social norms, potentially through direct precepts of pursuing a healthy life, or tenets that have an indirect effect on health.^7^ Religion, including the Islamic faith, can have a prohibitive influence against tobacco use and promote quitting among smokers.^10^, ^11^ Reinforcing health messages in interventions using Islamic scripture to change smoking behaviours has been reported as acceptable.^12^ Islamic faith-based teachings and teachers thus have a potential role in controlling tobacco use, and thereby reducing second-hand-smoke exposure in the home, but evidence on the effectiveness of faith-based interventions in changing smoking behaviours is scarce.^13^ Indoor-air-quality feedback, based on markers of second-hand smoke, such as the concentration of airborne particulate matter less than 2·5 microns (PM_2·5_) in diameter, can potentially motivate households to make their homes smoke-free.^14^ However, using indoor-air-quality feedback in this way is under-researched, particularly in LMICs.

We designed our interventions on the basis of theoretical work on the role of faith-based interventions to reduce smoking and the potential motivational effects of indoor-air-quality feedback.^10^, ^13^, ^14^ Our methods were based on a pilot trial done in England, which found that a smoke-free home intervention was acceptable to Muslim communities and feasible to be delivered through mosques.^15^ We designed a community-based smoke-free-home intervention in which religious leaders (ie, imams and khatibs) encouraged their mosque congregations to change their smoking behaviours. We aimed to evaluate the efficacy and cost-effectiveness of this community-based smoke-free-home intervention, with or without indoor-air-quality feedback, in reducing exposure to second-hand smoke in the home, frequency and severity of respiratory symptoms, and health service use, and in improving quality of life.

## Methods

### Study design and participants

We did a three-arm, open-label, cluster-randomised, controlled trial and cost-effectiveness analysis in which mosques in Mirpur, Dhaka, Bangladesh were recruited and households from their catchment communities enrolled (Muslim Communities Learning About Second-hand Smoke [MCLASS II] trial). The mosques were situated in residential areas of Dhaka, hosted regular communal prayers (including Friday Jumu'ah prayers), had a non-smoking religious leader (imam or khatib), and were affiliated with the Islamic Foundation under the Ministry of Religious Affairs, Bangladesh. Households (ie, single housing units shared by one or more people) were eligible if they had at least one resident attending one of the participating mosques, at least one adult resident (age 18 years or older) who smoked cigarettes or other forms of smoked tobacco (eg, bidi, waterpipe) regularly (on at least 25 days per month), and at least one non-smoking resident of any age. Households were excluded if they were planning to move home in the next 12 months, or if they used coal or biomass fuel for domestic cooking or heating. A resident was defined as an adult or child who had been living in the home for the preceding 3 months and planned to continue living in the household for at least 1 more year. Trial investigators recruited mosques by providing the mosque leaders with trial information and screening the mosques for eligibility. They also approached heads of household living in the catchment area and attending prayers at any of the participating mosques, either at the mosque or through a home visit, and provided them with study information; those interested were screened for eligibility.

Written informed consent was obtained from imams or khatibs for their and their mosques' participation, heads of household for participation of households, and adults in respective households for data collection and, if they are parents or guardians, for collecting data on their children. Ethics approval was obtained from the Bangladesh Medical Research Council's National Research Ethics Committee (BMBC/NREC/2016–2019/358) and the University of York's Health Sciences Research Governance Committee.

### Randomisation and masking

After recruitment and baseline data collection were completed, mosques and the consenting households from their congregation were centrally randomly assigned (1:1:1) to the smoke-free-home intervention plus indoor-air-quality feedback, smoke-free-home intervention only, or usual services. Minimisation, via MinimPY, was used to balance the average estimated size of the Friday Jumu'ah prayer congregation (≤1500 or >1500 people) and geographical location (wards within Mirpur). Randomisation was done by a statistician (CF) at the University of York, York, UK who was not involved in recruiting mosques or households. Mosques were input into MinimPY in a random order unknown to everyone except the statistician. Thus, even if the minimisation factors for the mosques were known, the allocations could not be predicted in advance, and allocation concealment was assured. Masking of participants and imams or khatibs was not possible. Outcome data collection and statistical analyses were also not masked.

### Procedures

The smoke-free-home intervention consisted of health messages relating to smoking and second-hand-smoke exposure, each supported by at least one Qur'an verse (ayah), or an Islamic faith-based decree. The messages were developed through iterative workshops involving Islamic scholars, public health professionals, and behavioural scientists. The messages were delivered by imams or khatibs to those attending Friday Jumu'ah prayer in mosques over 12 weeks (one message per week; see appendix 2 p 2 for examples). The messages addressed key determinants of current smoking behaviours including: poor knowledge on and attitudes towards smoking and second-hand-smoke exposure, by providing information on health consequences of smoking and second-hand-smoke exposure, including addressing misconceptions; and perceptions about social norms, by providing general information on others' approval. The messages also targeted prompting intentions, goal setting (both for behaviour [eg, attempting to quit] and the desired outcome of a smoke-free home), self-efficacy, commitment, action planning, coping planning, and sources of social support (appendix 2 p 22). Imams or khatibs in mosques that were randomly assigned to deliver the smoke-free-home intervention received an intervention booklet-based half-day training on the intervention and its delivery. They also received copies of the intervention booklet to distribute to their congregation members after Friday Jumu'ah prayers or in study circles. Intervention delivery started immediately after training and continued for 12 weeks.

Indoor-air-quality feedback comprised providing households with personalised information on the PM_2·5_ concentration measured within their home at baseline, in the form of a two-page bespoke leaflet, aimed at motivating changes in smoking behaviour in households. PM_2·5_ concentration was measured in homes using the Dylos DC 1700 (Dylos, Riverside, CA, USA), an optical particle counter validated for use in domestic settings.^16^ Feedback included a comparison of the household's mean PM_2·5_ concentration over 24 h with the WHO guidance limit^17^ of 25 μg/m^3^, the total time the household's PM_2·5_ concentration exceeded this guidance limit, and the maximum concentration measured in the household. Feedback also included pictorial information about the household's mean PM_2·5_ concentration (>150 μg/m^3^ was classified as hazardous, 36–150 μg/m^3^ as unhealthy, 12–35 μg/m^3^ as moderate, and <12 μg/m^3^ as good), information about adverse effects of second-hand-smoke exposure, recommendations to reduce second-hand-smoke exposure in the home, and a target air quality that was achievable by implementing smoke-free-home rules within the household. The leaflet was designed in consultation with lay community members. Trial investigators delivered and discussed the indoor-air-quality feedback with household members in person for approximately 10 min per household. After completing the final follow-up, all households in the three trial groups received feedback on indoor-air-quality measurements at 12 months after randomisation.

Households and mosques that were assigned to the usual services group received no intervention; however, following trial completion, mosques in the usual services group were offered the smoke-free-home intervention toolkit free of charge.

Data were collected at enrolment (baseline), and 3, 6, and 12 months after randomisation (appendix 2 p 2) using paper-based questionnaires administered by 16 investigators after they received 3 days training on trial procedures. Household air quality was measured at baseline, 3 months, and 12 months, by the same investigators. The data was entered into a password-protected database on a secure web application, Research Electronic Data Capture (REDCap). Further details on study design, participants, and procedures are provided in our published protocol.^18^

### Outcomes

The primary outcome was 24-h mean household PM_2·5_ concentration at 12 months after randomisation. Household-level secondary outcomes were: 24-h mean household PM_2·5_ concentration at 3 months after randomisation; and smoking restrictions at home, assessed through a questionnaire directed at adults in the households at 3, 6, and 12 months. Participant-level secondary outcomes assessed at each follow-up visit were: frequency and severity of respiratory symptoms, assessed using part one (eight questions) of the validated St George's Respiratory Questionnaire (SGRQ)^19^ for participants aged 11 years or older, and the severity scale developed and validated by Chauhan and colleagues^20^ for participants aged younger than 11 years; health-related quality of life, assessed using the EQ-5D-5L^21^ for adults (aged 18 years or older), EQ-5D-Y^22^ for adolescents (aged 11–17 years), and the paediatric quality of life inventory (PedsQL)^23^ for children (aged younger than 11 years); and health-care service use, measured using a questionnaire previously used in a pilot trial^15^ in England and adapted to the Bangladesh context.

### Statistical analysis

We planned to recruit 45 mosques and 40 households per mosque (n=1800), and to follow-up 30 households per mosque at 3 months after randomisation (n=1350), prioritising those with a baseline 24-h mean PM_2·5_ concentration of 35 μg/m^3^ or greater. Assuming an intracluster correlation coefficient of 0·02 and 20% attrition at 12 months, this would provide 90% power to detect an effect size of 0·3 SDs (equivalent to a difference of −13·5 μg/m^3^, from 76 μg/m^3^ to 62·5 μg/m^3^, assuming an SD of 45) for each pairwise comparison between groups, using a two-sided α of 0·05.

Analyses followed a prespecified analysis plan, approved by the trial steering committee before the completion of the 12-month data collection. No post-hoc analyses were done. All analyses used the intention-to-treat population and two-sided statistical tests at the 5% significance level in Stata version 15. Baseline and outcome data were summarised by trial group.

The primary analysis compared household 24-h mean PM_2·5_ concentrations between the groups using a covariance pattern, mixed-effect linear regression model incorporating the two post-randomisation timepoints (3 months and 12 months). The model included baseline PM_2·5_ concentration (household-level), geographical area, and size of Friday Jumu'ah prayer congregation in its continuous form (mosque-level), and timepoint, trial group, and a time-by-group interaction as fixed effects. Household and mosque were specified as random effects. An unstructured covariance pattern for the correlation of observations within households over time was specified, on the basis of minimising the Akaike information criterion. Visual inspection of model assumptions showed substantial deviations (appendix 2 pp 1, 23–24). Log-transformation of the outcome data improved model fit (appendix 2 pp 25–26) and was explored in sensitivity analyses. The pairwise mean differences between groups with 95% CI and p values at 3 and 12 months were extracted from the model.

The primary comparison was between the smoke-free-home intervention plus indoor-air-quality feedback group and usual services group at 12 months after randomisation. All other comparisons were secondary investigations. To account for non-compliance with trial group, we did a complier-average causal effect analysis^24^ for the primary outcome. A two-stage, least-squares instrumental variable approach was used, with trial group as the instrumental variable. Two analyses compared the 12-month outcome for each intervention with usual services. Within the smoke-free-home intervention only group, compliance was defined at the household-level as the lead adult reporting that they or another member of their household had received the smoke-free-home intervention from any mosque at any timepoint. Within the smoke-free-home intervention plus indoor-air-quality feedback group, compliance additionally included self-reported receipt of indoor-air-quality feedback by the 3-month follow-up.

Calibration of the Dylos machines before the 12-month follow-up indicated that they were consistently underestimating PM_2·5_ concentrations, relative to a gold-standard, factory-calibrated device, due to degradation of the laser particulate counter caused by heavy use at the baseline and 3-month assessments. This underestimation was corrected for in the primary analysis; details of sensitivity analyses assessing the effect of this correction are provided in the appendix 2 (p 1).

We did a subgroup analysis of whether any benefits from the interventions were greater among households with a baseline mean PM_2·5_ concentration of 35 μg/m^3^ or greater compared with households with a baseline concentration of less than 35 μg/m^3^, by including an interaction between dichotomised baseline PM_2·5_ concentration and trial group in the primary analysis.

Participant-level respiratory symptom scores were analysed in an analogous way to the primary outcome. Participant, household, and mosque were nested random effects. Analyses were done separately for the SGRQ symptoms component score for adults, for adolescents aged 11–17 years, and for the total symptoms severity scale for children aged younger than 11 years. Since both these instruments measure the same construct (respiratory symptoms), we did an additional analysis that included all participants using standardised scores. Model assumptions were assessed as for the primary analysis; no major deviations were observed, so data transformation was unnecessary.

We did a within-trial cost-effectiveness analysis comparing the smoke-free-home intervention, with and without indoor-air-quality feedback, versus usual services. The analysis used a health-care sector and intervention-provider perspective to include health-care resource use and intervention delivery costs. No discounting was applied as the follow-up period was 12 months.

All costs were calculated using a bottom-up approach. Costs for training staff to deliver the intervention (eg, teaching materials, support) were estimated on the basis of the cost incurred alongside the trial, whereas information on health-care resource use (eg, number of inpatient stays, outpatient visits) was collected from participants. The unit costs of home visits by doctors or nurses were obtained from the Bangladesh Bureau of Statistics,^25^ costs of inpatient stays and outpatient visits were derived from WHO's Bangladesh-specific unit costs,^26^ and costs of emergency department visits were extracted from the Bangladesh essential health service package (table 1).^27^ All costs were expressed in 2018–19 US$, using the 2018 World Development Indicators exchange rates.^28^

**Table 1:** Cost breakdown for interventions and health care

		**Unit cost (US$)**	**Source**
**Intervention costs**			
Training cost for indoor-air-quality feedback			
	Trainers (staff time)	39·4 per day	Trial team
	Trainees (staff time)	12·2 per day	Trial team
	Materials	2·7 per person	Trial team
	Logistics	1·0 per person	Trial team
	Venue	48·8	Trial team
Training cost for smoke-free-home intervention			
	Trainers (staff time)	4·9 per h	Trial team
	Trainees (staff time)	3·7 per h	Trial team
	Food	3·1 per person	Trial team
	Travel	640·0	Trial team
	Venue	48·8	Trial team
Delivery cost for indoor-air-quality feedback			
	Adaptor	30·75 each	Trial team
	Battery	332·5 each	Trial team
	Shipping cost	1046·3	Trial team
	Tax at airport	9648·3	Trial team
	Staff time	12·2 per day	Trial team
	Travel cost	1·2 per visit	Trial team
	Consumables (eg, booklets, food)	219·8	Trial team
Delivery cost for smoke-free-home intervention			
	Religious leaders (staff time)	3·7 per h	Trial team
	Consumables	0·4 per copy	Trial team
	Booklets	4·9 per copy	Trial team
**Health-care costs**			
Inpatient stays		54·4 per stay	WHO^26^
Outpatient visits		1·9 per visit	WHO^26^
Emergency department visits		2·5 per visit	Islam and colleagues^27^
Home visits (doctor)*		13·5 per visit	Bangladesh Bureau of Statistics^25^
Home visits (nurse)*		8·5 per visit	Bangladesh Bureau of Statistics^25^

* The average time for a home visit was assumed to be 1 h 40 min, including travel time.

Due to the absence of unified and established tariffs from Bangladesh for the three instruments used to measure health-related quality of life (ie, PedsQL, EQ-5D-Y, and EQ-5D-5L), relevant UK value sets were used and mapped to the corresponding EQ-5D-3L values,^23^, ^29^ which allowed us to obtain unified utility estimates across individuals. A sensitivity analysis using Thailand value sets^30^ for EQ-5D-Y and EQ-5D-5L was done to test the robustness of the results. Quality-adjusted life-years (QALYs) for individuals were calculated using the area under the curve method over the trial period.^31^

Cost-effectiveness was evaluated using the pairwise incremental cost-effectiveness ratios (ICERs) method^31^ at a household level and assessed on the basis of the Bangladesh willingness-to-pay threshold: $30–427 per QALY gained.^32^ Seemingly unrelated regression was used to account for potential correlations between costs and QALYs, and to adjust for prognostic baseline covariates.^33^ Uncertainty was estimated using the non-parametric bootstrapping technique, with 5000 replications that were presented on a cost-effectiveness plane.^31^

### Role of the funding source

The funder of the study had no role in study design, data collection, data analysis, data interpretation, or writing of the report.

## Results

116 mosques were assessed for eligibility and 45 were recruited (figure). Reasons for exclusion were: being less than 0·5 km from another mosque participating in the trial (n=54), imams or khatibs not providing consent to participate (n=7), mosque catchment area having entry restrictions (n=7), small catchment area (n=2), and not performing Friday Jumu'ah prayers (n=1). Between April 11 and Aug 2, 2018, 4430 households were screened for eligibility; 1801 (40·7%) were eligible and enrolled. For the households that were ineligible, the reasons are provided in appendix 2 (p 2). Every mosque recruited 40 households, except one (allocated to usual services), which recruited 41. 16 mosques (640 households [35·5%]) were randomised to the smoke-free-home intervention plus indoor-air-quality feedback, 14 (560 [31·1%]) to the smoke-free-home intervention only, and 15 (601 [33·4%]) to usual services (appendix 2 pp 3–4).

**Figure:**
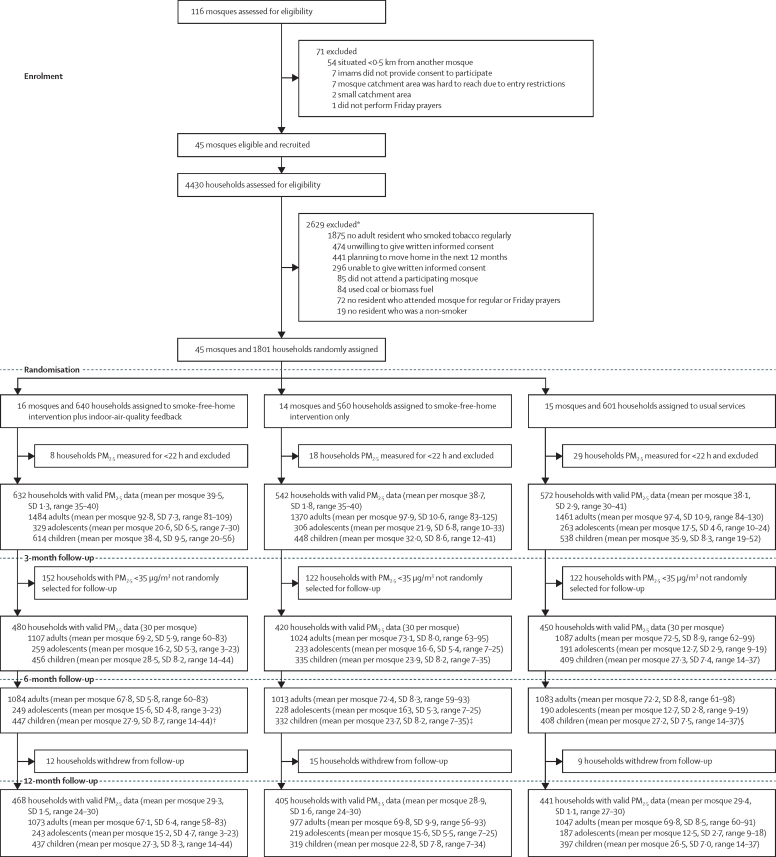
Trial profile Adults were those aged 18 years or older, adolescents were those aged 11–17 years, and children were those aged younger than 11 years. PM_2·5_=airborne particulate matter less than 2·5 microns in diameter. *Some households met more than one exclusion criterion. †Data from 472 households. ‡Data from 418 households. §Data from 450 households.

Of the 712 households with mean baseline PM_2·5_ concentration of 35 μg/m^3^ or greater, 614 (86·2%) were followed-up at 3 months; 98 (13·8%) had either moved away from the study area or did not wish to continue the study. To achieve the target of 1350 households followed-up at 3 months, we randomly selected another 736 households with mean baseline PM_2·5_ concentration less than 35 μg/m^3^ to follow-up, as per protocol (table 2, appendix 2 pp 4–11). Of these 1350 households, 1314 (97·3%) completed follow-up again at 12 months (2·7% attrition rate; 2·0% in the usual services group, 3·6% in the smoke-free-home intervention only group, and 2·5% in the smoke-free-home intervention plus indoor-air-quality feedback group).

**Table 2:** Baseline characteristics of households (as followed-up)

		**Usual services group (n=450)**	**Smoke-free-home intervention only group (n=420)**	**Smoke-free-home intervention plus indoor-air-quality feedback group (n=480)**	**Total (n=1350)**
Age of head of household, years					
	Mean (SD)	40·8 (12·8)	40·7 (12·7)	40·3 (12·1)	40·6 (12·5)
	Median (range)	38·2 (20·1–85·4)	37·3 (22·1–85·5)	38·1 (20·2–84·7)	38·1 (20·1–85·5)
Gender of head of household					
	Male	429 (95·3%)	387 (92·1%)	461 (96·0%)	1277 (94·6%)
	Female	21 (4·7%)	33 (7·9%)	19 (4·0%)	73 (5·4%)
Number of adult residents					
	Mean (SD)	2·4 (0·8)	2·5 (0·8)	2·3 (0·7)	2·4 (0·8)
	Median (range)	2·0 (1·0–6·0)	2·0 (1·0–6·0)	2·0 (1·0–5·0)	2·0 (1·0–6·0)
Number of child residents					
	Mean (SD)	1·3 (1·1)	1·4 (1·1)	1·5 (1·1)	1·4 (1·1)
	Median (range)	1·0 (0·0–7·0)	1·0 (0·0–5·0)	1·0 (0·0–6·0)	1·0 (0·0–7·0)
Homes with outside space		244 (54·2%)	243 (57·9%)	237 (49·4%)	724 (53·6%)
Number of bedrooms					
	Mean (SD)	1·5 (0·7)	1·5 (0·7)	1·3 (0·6)	1·4 (0·7)
	Median (range)	1·0 (0·0–5·0)	1·0 (1·0–5·0)	1·0 (1·0–4·0)	1·0 (0·0–5·0)
Type of fuel used for cooking					
	Electricity	41 (9·1%)	71 (16·9%)	11 (2·3%)	123 (9·1%)
	LPG, natural gas, or biogas	401 (89·1%)	362 (86·2%)	479 (99·8%)	1242 (92·0%)
	Kerosene	27 (6·0%)	5 (1·2%)	21 (4·4%)	53 (3·9%)
Number of adult resident smokers					
	Mean (SD)	1·1 (0·3)	1·1 (0·3)	1·1 (0·3)	1·1 (0·3)
	Median (range)	1·0 (1·0–3·0)	1·0 (0·0–3·0)	1·0 (0·0–3·0)	1·0 (0·0–3·0)
Number of child resident smokers					
	Mean (SD)	0·0 (0·1)	0·0 (0·1)	0·0 (0·2)	0·0 (0·1)
	Median (range)	0·0 (0·0–1·0)	0·0 (0·0–1·0)	0·0 (0·0–1·0)	0·0 (0·0–1·0)
Residents allowed to smoke					
	Anywhere inside the home	197 (43·8%)	180 (42·9%)	262 (54·6%)	639 (47·3%)
	Only in some rooms in the home	5 (1·1%)	6 (1·4%)	1 (0·2%)	12 (0·9%)
	Only in one room in the home	14 (3·1%)	36 (8·6%)	15 (3·1%)	65 (4·8%)
	Only outside	233 (51·8%)	196 (46·7%)	199 (41·5%)	628 (46·5%)
	Not known	1 (0·2%)	2 (0·5%)	3 (0·6%)	6 (0·4%)
Visitors allowed to smoke					
	Anywhere inside the home	178 (39·6%)	146 (34·8%)	247 (51·5%)	571 (42·3%)
	Only in some rooms in the home	4 (0·9%)	6 (1·4%)	1 (0·2%)	11 (0·8%)
	Only in one room in the home	11 (2·4%)	30 (7·1%)	12 (2·5%)	53 (3·9%)
	Only outside	250 (55·6%)	229 (54·5%)	213 (44·4%)	692 (51·3%)
	Not known	7 (1·6%)	9 (2·1%)	7 (1·5%)	23 (1·7%)
Residents allowed to smoke in front of children in the home					
	Yes	132 (29·3%)	142 (33·8%)	194 (40·4%)	468 (34·7%)
	No	221 (49·1%)	179 (42·6%)	191 (39·8%)	591 (43·8%)
	Not known	6 (1·3%)	3 (0·7%)	10 (2·1%)	19 (1·4%)
	No child residents	91 (20·2%)	96 (22·9%)	85 (17·7%)	272 (20·1%)
Visitors allowed to smoke in front of children in the home					
	Yes	118 (26·2%)	112 (26·7%)	196 (40·8%)	426 (31·6%)
	No	234 (52·0%)	201 (47·9%)	185 (38·5%)	620 (45·9%)
	Not known	9 (2·0%)	12 (2·9%)	14 (2·9%)	35 (2·6%)
	No child residents	89 (19·8%)	95 (22·6%)	85 (17·7%)	269 (19·9%)

Data are n (%) unless otherwise stated. Adults were defined as those aged 18 years or older, children were those aged younger than 18 years. LPG=liquified petroleum gas.

At 12 months follow-up, the mean 24-h PM_2·5_ concentration was 65·8 μg/m^3^ (SD 39·6) in the smoke-free-home intervention plus indoor-air-quality feedback group, 68·9 μg/m^3^ (49·5) in the smoke-free-home intervention only group, and 65·2 μg/m^3^ (44·7) in the usual services group (table 3). No evidence of a difference was observed at 12 months for any pairwise comparison, including when the outcome data were log-transformed (table 4, appendix 2 p 26). The adjusted mean differences in PM_2·5_ concentration were −1·0 μg/m^3^ (95% CI −12·8 to 10·9) for the smoke-free-home intervention plus indoor-air-quality feedback group versus usual services group (p=0·88); 5·0 μg/m^3^ (–7·9 to 18·0) for the smoke-free-home intervention only group versus usual services group (p=0·45); and −6·0 μg/m^3^ (–18·3 to 6·3) for the smoke-free-home intervention plus indoor-air-quality feedback group versus the smoke-free-home intervention only group (p=0·34). The log-transformed sensitivity analysis indicated that the mean PM_2·5_ concentrations in the smoke-free-home intervention plus indoor-air-quality feedback group were expected to be 1·02 times larger (95% CI 0·86 to 1·21; p=0·79) than the usual services group. The estimated mosque-level intracluster correlation coefficient was 0·08 (95% CI 0·05 to 0·14).

**Table 3:** Household PM_2·5_ concentrations by trial group over time

	**Usual services group (n=601)**	**Smoke-free-home intervention only group (n=560)**	**Smoke-free-home intervention plus indoor-air-quality feedback group (n=640)**	**Total (n=1801)**
**Baseline (as randomised)**				
Households, n	572	542	632	1746
Mean (SD)	41·9 (38·5)	39·5 (29·3)	44·6 (43·7)	42·2 (38·0)
Median (range)	29 (2–251)	30 (8–166)	30 (1–422)	30 (1–422)
**Baseline (as analysed)**				
Households, n	447	419	480	1346
Mean (SD)	44·2 (40·8)	42·1 (30·7)	46·6 (42·3)	44·4 (38·6)
Median (range)	30 (2–251)	34 (8–166)	30 (5–334)	31 (2–334)
**3 months**				
Households, n	450	420	480	1350
Mean (SD)	85·3 (86·8)	75·6 (83·7)	74·9 (76·9)	78·6 (82·5)
Median (range)	44·5 (1–417)	38 (6–459)	38 (1–353)	39 (1–459)
**12 months**				
Households, n	441	405	468	1314
Mean (SD)	65·2 (44·7)	68·9 (49·5)	65·8 (39·6)	66·5 (44·6)
Median (range)	54 (11–340)	57 (13–389)	56 (14–244)	55 (11–389)

PM_2·5_ concentrations are in μg/m^3^. PM_2·5_=airborne particulate matter less than 2·5 microns in diameter.

**Table 4:** Differences in household PM_2·5_ concentration over time between trial groups, presented for both raw and log-transformed outcome data

		**Adjusted mean difference in PM**_2·5_**concentration (95% CI)**	**p value**
**Primary analysis**			
3 months			
	Smoke-free-home intervention plus indoor-air-quality feedback *vs* usual services	−12·6 (−26·3 to 1·0)	0·070
	Smoke-free-home intervention only *vs* usual services	−9·3 (−24·0 to 5·4)	0·22
	Smoke-free-home intervention only *vs* smoke-free-home intervention plus indoor-air-quality feedback	3·4 (−10·7 to 17·4)	0·64
12 months			
	Smoke-free-home intervention plus indoor-air-quality feedback *vs* usual services	−1·0 (−12·8 to 10·9)	0·88
	Smoke-free-home intervention only *vs* usual services	5·0 (−7·9 to 18·0)	0·45
	Smoke-free-home intervention only *vs* smoke-free-home intervention plus indoor-air-quality feedback	6·0 (−6·3 to 18·3)	0·34
**Log-transformed (sensitivity analysis)**			
3 months			
	Smoke-free-home intervention plus indoor-air-quality feedback *vs* usual services	−0·13 (−0·32 to 0·05)	0·16
	Smoke-free-home intervention only *vs* usual services	−0·11 (−0·30 to 0·09)	0·30
	Smoke-free-home intervention only *vs* smoke-free-home intervention plus indoor-air-quality feedback	0·03 (−0·16 to 0·22)	0·78
12 months			
	Smoke-free-home intervention plus indoor-air-quality feedback *vs* usual services	0·02 (−0·15 to 0·19)	0·79
	Smoke-free-home intervention only *vs* usual services	0·06 (−0·12 to 0·25)	0·50
	Smoke-free-home intervention only *vs* smoke-free-home intervention plus indoor-air-quality feedback	0·04 (−0·13 to 0·21)	0·64

PM_2·5_ concentrations are in μg/m^3^. PM_2·5_=airborne particulate matter less than 2·5 microns in diameter.

At 3-months follow-up, there was no evidence of a difference in the mean 24-h PM_2·5_ concentrations for any pairwise comparison, including when the outcome data were log-transformed (table 4; appendix 2 p 26). There was evidence of small differences in some secondary comparisons between the smoke-free-home intervention only group and the smoke-free-home intervention plus indoor-air-quality feedback group, favouring intervention plus feedback (SGRQ for adults, at 6 months: adjusted mean difference in outcome score 2·4 [95% CI 0·3–4·6], p=0·028; respiratory symptoms in children aged younger than 11 years, at 3 months: 2·0 [0·5–3·4], p=0·0083; standardised respiratory scores in all participants, at 6 months: 0·14 [0·00–0·29], p=0·044). No other differences were observed at 3 months, 6 months, or 12 months (appendix 2 pp 12–17).

22·9% of households (usual services group n=110 [24·9%]; smoke-free-home intervention only group n=76 [18·8%]; smoke-free-home intervention plus indoor-air-quality feedback group n=115 [26·6%]) reported at 12 months that residents were permitted to smoke anywhere inside the home (appendix 2 p 17).

In the sensitivity analysis, we found that 331 (78·8%) of 420 households in the smoke-free-home intervention only group received the intervention; in addition, 91 (20·2%) of 450 households in the usual services group reported receiving some element of the intervention; the complier-average causal effect estimate after receiving the smoke-free-home intervention was an increase in mean PM_2·5_ concentration of 11·3 μg/m^3^ (95% CI −6·7 to 29·2). In the smoke-free-home intervention plus indoor-air-quality feedback group, 351 (73·1%) of 480 households received the intervention and feedback by the 3-month follow-up; the complier-average causal effect estimate after receiving the intervention and indoor-air-quality feedback was a decrease in PM_2·5_ concentration (–3·0 μg/m^3^ [–17·4 to 11·4]). No differences were observed at 3 months or 12 months in the other sensitivity analyses (appendix 2 pp 18–19, 27–28).

In the subgroup analysis, we found no evidence of an interaction with baseline PM_2·5_ concentration (<35 μg/m^3^ compared with ≥35 μg/m^3^; appendix 2 pp 20, 29–30).

From the 1350 households followed-up at 3 months, 4893 (95·1%) of 5143 participants had complete cost and QALY data at all follow-ups. After removing households with participants with incomplete data, 1237 (91·6%) of 1350 households were included in the cost-effectiveness analysis. The smoke-free-home intervention plus indoor-air-quality feedback group incurred the highest mean total cost ($32·8 [SD 22·0]) and generated the highest mean QALYs (3·31 [SD 1·20]; table 5). The smoke-free-home intervention only group incurred higher costs but generated less QALYs compared with the usual services group, and was therefore dominated. Due to high delivery cost of indoor-air-quality feedback ($16·1), intervention cost was the key cost driver for the smoke-free-home intervention plus indoor-air-quality feedback group, but not for the smoke-free-home intervention only group (appendix 2 p 20). The smoke-free-home intervention plus indoor-air-quality feedback was not cost-effective, as the ICER of $653 per QALY compared with usual services was more than the willingness-to-pay threshold of $30–427 per QALY gained. The results of the 5000 bootstrapped seemingly unrelated regression models are shown in table 5 and appendix 2 (p 31). The bootstrapped ICERs were within the top-left quadrant of the cost-effectiveness plane and above the willingness-to-pay threshold lines, indicating that both the intervention only, and the combination of the intervention plus indoor-air-quality feedback, were not cost-effective, even when taking uncertainly into consideration. The results of the sensitivity analysis were similar to the main findings (appendix 2 pp 21, 32).

**Table 5:** Costs, QALYs, and ICERs by trial group

	**Households, n**	**Costs**			**QALYs (SD)**	**ICER**
		Intervention, US$	Health care, US$ (SD)	Total US$ (SD)		
**Trial group**						
Smoke-free-home intervention plus indoor-air-quality feedback	429	21·9	11·0 (22·0)	32·8 (22·0)	3·31 (1·20)	$653 per QALY gained
Smoke-free-home intervention only	383	2·9	23·0 (61·0)	25·8 (61·0)	3·25 (1·18)	Dominated
Usual services	425	0	13·2 (30·4)	13·2 (30·4)	3·28 (1·22)	Reference
**Bootstrapped seemingly unrelated regression models**						
Smoke-free-home intervention plus indoor-air-quality feedback *vs* usual services	..	..	..	19·5 (14·2 to 24·9)	−0·05 (−0·05 to 0·05)	Dominated
Smoke-free-home intervention only *vs* usual services	..	..	..	12·1 (6·6 to 17·6)	−0·10 (−0·14 to −0·07)	Dominated

Costs and QALYs for bootstrapped seemingly unrelated regression models are incremental means with 95% CI. QALY=quality-adjusted life-year. ICER=incremental cost-effectiveness ratio.

## Discussion

The smoke-free-home intervention, with or without indoor-air-quality feedback, did not reduce exposure to second-hand smoke in the home, measured as the mean 24-h PM_2·5_ concentration within households, compared with usual services. Our cost-effectiveness analysis suggests that both the smoke-free-home intervention only and the combination of the smoke-free-home intervention and indoor-air-quality feedback were not cost-effective compared with usual services, due to high intervention costs and minimal QALY gains.

To our knowledge, this trial is the first to investigate the efficacy and cost-effectiveness of community-based interventions, delivered within an Islamic discourse by religious leaders in mosques, to reduce second-hand-smoke exposure within the home. We found that it is feasible and acceptable to do large studies of such interventions within mosques. The trial is also the largest of its kind to provide 24-h household-level PM_2·5_ concentration data, and explore the usefulness of using indoor-air-quality feedback as a motivational tool for reducing second-hand-smoke exposure in the home, in a LMIC setting. Other strengths of the trial were the rigour and quality with which it was done; the cluster-randomised, controlled design; achieving the required sample size; high follow-up rates; high levels of data completeness; and the 12-month follow-up duration.

There are several potential explanations for the absence of effectiveness of the interventions. Sermons where the intervention messages were delivered are not mandatory, and therefore some people might have joined the prayers, but not attended or paid the desired level of attention to the sermons. Although intervention compliance as defined in our trial was high, individuals might have received some, but not all, messages. Because of the nature of the intervention, it was not possible to calculate the so-called dose of the intervention received by household members. The interventions targeted reducing second-hand-smoke exposure in the home directly, and did not offer smoking cessation support to smokers within the home. Aspirations to make homes smoke-free might have been constrained by the scarcity of social and environmental opportunities to change behaviour.^34^, ^35^ Thus, a standalone community-based intervention delivered over a short period might have been insufficient to change smoking behaviours in Bangladesh, where regulatory and fiscal measures for tobacco control are weak, cigarettes and bidis are cheap, and smoking cessation services are scarce. In addition, the personalised indoor-air-quality feedback was delayed, due to the need to take the Dylos machine back to the office to download the data and generate the graphical and numerical feedback, and was not targeted specifically at smokers.

The intervention effects on PM_2·5_ concentration in the home could have been diluted due to a Hawthorne effect across all trial groups during the baseline 24-h measurement period when the Dylos devices were present in the home.^36^ Members of the household, particularly smokers, might have modified their behaviour (perhaps by reducing smoking inside the house or near the Dylos machine) in response to being aware that the device was recording the air quality in their home. Measuring PM_2·5_ concentrations over a longer period could have reduced this potential bias by making it more difficult to sustain behavioural change over the whole measurement period.^36^ However, this would have been more costly due to the need for more devices.

PM_2·5_ concentration is not specific to tobacco smoke; it can also be generated by non-tobacco sources such as using solid fuels and vehicle and industrial emissions. We addressed other PM_2.5_ concentration influences by excluding households that used coal or biomass fuel for domestic use and restricting measurements to the period of April–October, when outdoor air pollution levels in Dhaka are at their lowest.^37^ We also used a cluster-randomised, controlled design to balance such confounders between the two groups. Therefore, any change observed in the primary outcome between the two groups would have been most likely due to change in smoking behaviour. Confidence in our findings is also enhanced by the fact that baseline PM_2.5_ concentrations were significantly lower in smoke-free homes when compared with homes where smoking was permitted, despite high ambient air pollution.^38^

Participants' health-related quality of life was measured using three different instruments (the EQ-5D-5L for adults, EQ-5D-Y for adolescents, and PedsQL for children) due to the absence of a universal instrument that could measure health-related quality of life across all age groups. As reported, health-related quality of life might differ depending on which instrument is being used, this approach can result in household QALY estimations being sensitive to the number of people and the age composition in each household. However, this effect is unlikely to have affected our conclusion, as we controlled for household composition in the analysis. As there were no established Bangladesh population tariffs, the UK population tariffs were used for QALY calculations, as they are the only tariffs that can convert all three instrument measurements into consistent EQ-5D-3L values. Future studies on Bangladesh tariffs, and for other LMICs, across all age groups, are required to obtain more precise estimates.

With regards to faith-based behavioural change interventions, our findings can be generalised to other community-based interventions that are delivered primarily through mosques. When considering indoor-air-quality feedback, our study findings can be generalised to other urban centres similar to Dhaka, with high population density, high levels of ambient air pollution, and little opportunity to smoke outside.

Contrary to our findings, studies in other areas such as cardiovascular diseases, obesity, and breast cancer screening have suggested that health programmes delivered through faith-based organisations can improve outcomes.^8^, ^9^ Islamic faith-based smoking cessation interventions have also been found to be effective in encouraging Muslim smokers to stop smoking during Ramadan, although the sustainability of the behavioural change is unclear.^12^, ^39^ Nevertheless, our findings are consistent with those from other studies targeting reduction of second-hand-smoke exposure within the home using behavioural interventions and indoor-air-quality feedback. A review from 2018 found that the effectiveness of several counselling and educational interventions that have been used to try to reduce second-hand-smoke exposure has not been clearly shown.^40^ More successful interventions seem to be those that combine smoke-free-home interventions with smoking cessation advice and support for smokers within the home, or those that target smoking cessation as a pathway to reducing second-hand-smoke exposure.^41^ Additionally, a study from 2020 showed that real-time particle feedback and coaching contingencies reduced indoor air pollution from behaviours such as smoking cigarettes or burning candles.^42^ Hence, future research in LMICs should investigate the effectiveness of interventions that include offering smoking cessation to smokers within the household, and measures that offer real-time or immediate, rather than delayed, feedback on indoor air quality. Nevertheless, these technologies need to be low cost if they are to be cost-effective and scalable in LMICs.

## Data sharing

De-identified individual participant data on which summary statistics and tables are based will be made available from the point of, and up to 5 years after the, acceptance for publication of the main findings from the final dataset. These data can be requested from the Principal Investigator (Prof Kamran Siddiqi; kamran.siddiqi@york.ac.uk) and will be shared after the provision of a methodologically sound proposal, and only under a data-sharing agreement that provides for commitment to: using the data only for research purposes and not to identify any individual participant; securing the data using appropriate computer technology; and destroying or returning the data after analyses are completed. The proposals will be assessed and approved by members of the Programme Management Group. The intervention manual and indoor-air-quality feedback leaflet are available on the study webpage: https://www.york.ac.uk/healthsciences/research/public-health/projects/mclass11/#tab-3. Other materials such as participant information sheets, informed consent forms, and questionnaires will be made freely available to anyone who wishes to access them from the point of, and up to 5 years after the, acceptance for publication of the main findings from the trial. Requests can be made to the Principal Investigator.

## Supplementary Materials

Bengali translation of the Abstract
