# Supplementary material for: Efficacy and cost-effectiveness of a community-based smoke-free-home intervention with or without indoor-air-quality feedback in Bangladesh (MCLASS II): a three-arm, cluster-randomised, controlled trial
Source: Lancet Glob Health. 2021 Apr 15;9(5):e639–50. doi: 10.1016/S2214-109X(21)00040-1 (PMC8064237; doi:10.1016/S2214-109X(21)00040-1)
Supplement: Bengali translation of the Abstract [file mmc1.pdf]

THE LANCET
Global Health

Supplementary appendix 1

This translation in Bengali was submitted by the authors and we reproduce it as supplied. It has not been peer reviewed. *The Lancet*'s editorial processes have only been applied to the original in English, which should serve as reference for this manuscript.

'এই [বাংলায়] অনুবাদটি লেখকরা জমা দিয়েছিলেন এবং এটি যেমনভাবে দেওয়া হয়েছে আমরা সেইভাবেই পুনরায় বর্ণনা করছি। এটি কোনো সমকক্ষ ব্যক্তি পর্যালোচনা করেননি। দ্য ল্যানসেট–এর সম্পাদকীয় প্রক্রিয়াগুলি শুধুমাত্র মূল ইংরেজিতে প্রয়োগ করা হয়েছে, যা এই পান্ডুলিপির রেফারেন্স হিসাবে কাজ করবে।'

Supplement to: Mdege ND, Fairhurst C, Wang H-I, et al. Efficacy and cost-effectiveness of a community-based smoke-free-home intervention with or without indoor-air-quality feedback in Bangladesh (MCLASS II): a three-arm, cluster-randomised, controlled trial. *Lancet Glob Health* 2021; **9:** e639–50.

APPENDIX 1

বাংলাদেশে ‘ধূমপানমুক্ত বাড়ি’ নামক কমিউনিটি ভিত্তিক ইন্টারভেনশন (MCLASS II) এর কার্যকারীতা এবং কার্যকারিতা সংক্রান্ত ব্যায় (Cost effectiveness): একটি থ্রি-আর্ম, ক্লাস্টার র‍্যান্ডোমাইজড কন্টোলড ট্রায়াল

নরীন ডাডিরাই মেজ, ক্যারোলিন ফেয়ারহার্স্ট, হান-ই ওয়াং, তারানা ফেরদৌস, আনা-মেরি মার্শাল, ক্যাথেরিন হিউইট, রুমানা হক, ক্যাথ জ্যাকসন, ইয়ান কেলার, স্টীভ প্যারট, শন সেম্পল, আজিজ শেখ, কীই-উ, জুনায়েদ আল আজদী, কামরান সিদ্দিকী, MCLASS II ট্রায়াল টীমের পক্ষ থেকে

সার-সংক্ষেপঃ

পটভূমিঃ বিশ্বব্যাপী রোগাক্রান্তের সংখ্যা এবং প্রাণহানীর ক্ষেত্রে একটি প্রধান কারন হচ্ছে তামাকের ধোঁয়া দ্বারা মানুষের থেকে পরোক্ষ ধূমপানের শিকার হওয়া।
বাংলাদেশে ঘরের অভ্যন্তরে মানুষকে পরোক্ষ ধূমপানের শিকার হওয়া কমিয়ে আনার জন্য আমরা ‘ধূমপানমুক্ত বাড়ি’ নামক কমিউনিটি ভিত্তিক ইন্টারভেনশন, এবং একই সাথে গৃহাভ্যন্তরীন বাতাসের গুণগত মানের ফলাফল সহ কিংবা এই ফলাফল ব্যাতিরেকে-এর ফলপ্রসূতা এবং কার্যকারিতা সংক্রান্ত ব্যায় (cost effectiveness) এর মূল্যায়ন করার কাজ করেছি।

গবেষণা-পদ্ধতিঃ আমরা বাংলাদেশে একটি থ্রি-আর্ম, ক্লাস্টার র‍্যান্ডোমাইজড কন্টোলড ট্রায়াল পরিচালনা করেছি। এর আওতায় আমরা নির্দিষ্ট এলাকার কিছু মসজিদ দৈবচয়ন ভিত্তিতে (র‍্যান্ডমলি) এবং ১:১:১ অনুপাতে তিনটি শাখায় (আর্ম-এ) অন্তর্ভুক্ত করেছি এবং উক্ত মসজিদসমূহে জামাতে অংশগ্রহনকারী ব্যাক্তিদের ঘর (পরিবার) সমূহকে তাঁদের সম্মতিক্রমে অন্তর্ভুক্ত করেছি; অন্তর্ভুক্ত ঘরের মাঝে একটি দল ‘ধূমপানমুক্ত বাড়ি’ নামক ইন্টারভেনশন গ্রহন করেছেন ও সেই সাথে তাঁদেরকে গৃহাভ্যন্তরীন বাতাসের গুণগত মান সংক্রান্ত ফলাফল জানান হয়েছে; আরেকটি দলের অন্তর্ভুক্ত পরিবার কেবলমাত্র ‘ধূমপানমুক্ত বাড়ি’ নামক ইন্টারভেনশন গ্রহন করেছেন; এবং অবশিষ্ট দলে অংশগ্রহনকারীরা কন্ট্রোল আর্ম হিসেবে গতানুগতিক সেবা (usual services) গ্রহন করেছেন। উক্ত ঘরগুলো কিছু বৈশিষ্ট্যের সাপেক্ষে গবেষণার উপযুক্ত বলে বিবেচিত হয়েছে এবং প্রকল্পে অন্তর্ভুক্ত হয়েছে। বৈশিষ্ট্যগুলো হচ্ছে - ঘরের অন্ততঃ একজন গবেষণায় অন্তর্ভুক্ত মসজিদের জামাতে অংশগ্রহন করে থাকেন, এবং উক্ত ঘরের কমপক্ষে একজন প্রাপ্তবয়স্ক সদস্য (১৮ কিংবা এর বেশী বয়সী) নিয়মিতভাবে (প্রতি মাসে কমপক্ষে ২৫ দিন কিংবা এর চেয়েও বেশি দিন ধরে) সিগারেট খেয়ে থাকেন কিংবা ধোঁয়াযুক্ত তামাকের অন্য যেকোনো পণ্য (বিড়ি, হুক্কা) ব্যবহার করে ধুমপান করে থাকেন; এবং উক্ত গৃহে কমপক্ষে একজন যেকোনো বয়সী অধূমপায়ী বসবাস করেন।

‘ধূমপানমুক্ত বাড়ি’ নামক ইন্টারভেনশন কার্যক্রমটি হচ্ছেঃ মোট ১২ সপ্তাহ সময় যাবৎ, মসজিদের ইমাম/খতিবের দ্বারা প্রতি সপ্তাহের জুম্মার নামাযের খুতবায় স্বাস্থ্য সংক্রান্ত বার্তা প্রদান করা।

গৃহাভ্যন্তরীন বাতাসের গুণগত মান সংক্রান্ত ফলাফল বলতে বোঝানো হয়েছে যে, প্রতিটি গৃহে প্রতি ২৪ ঘন্টায় অভ্যন্তরীন বাতাসের গুণগত মানের বিষয়ে উক্ত পরিবারকে অবহিত করা।

গতানুগতিক সেবা (usual services) –এর অন্তর্ভুক্ত দলটিকে কোনো ইন্টারভেনশন প্রদান করা হয়নি।

অংশগ্রহনকারী পরিবার এবং মসজিদের ইমাম বা খতিবের নিকট তাঁদের গবেষণায় অন্তর্ভুক্তির শাখা (আর্ম) সংক্রান্ত বিবরণ (অর্থাৎ তাঁরা ইন্টারভেনশন পাবেন কি-না সেই তথ্য) আড়াল করে রাখা (মাস্কিং) সম্ভব হয়নি।

র‍্যান্ডোমাইজেশনের ১২ মাস পর প্রতি ২৪ ঘন্টায় গৃহাভ্যন্তরীন বাতাসে উপস্থিত সূক্ষ্ম কণা (যার ব্যাস ২.৫ মাইক্রনের চেয়ে কম অর্থাৎ বস্তুকণা বা পার্টিকুলেট ম্যাটার ২.৫) –এর গড় ঘনত্বের পরিমাণ (যেটি দ্বারা পরোক্ষ ধূমপান হওয়ার বিষয়টি চিহ্নিত করা যায়) থেকে প্রাথমিক ফলাফল নির্নীত হয়েছিল।

ক্রমবর্ধমান কার্যকারিতা সংক্রান্ত ব্যায় এর অনুপাত (incremental cost effectiveness ratio) ব্যবহার করে কার্যকারিতা সংক্রান্ত ব্যায় (cost effectiveness) এর বিষয়টি মূল্যায়ন করা হয়েছে।

এই ট্রায়ালটি ইন্টারন্যাশনাল স্ট্যান্ডার্ড র‍্যান্ডোমাইজড কন্ট্রোল ট্রায়াল নাম্বার (ISRCTN) দ্বারা নিবন্ধিত, যেটির নিবন্ধন সংখ্যা হচ্ছে ৪৯৯৭৫৪৫২।

ফলাফলঃ ২০১৮ সালের ১১ই এপ্রিল থেকে ২রা আগস্ট পর্যন্ত, আমরা ৪৫টি মসজিদের আওতায় ১৮০১টি খানা/ঘর (পরিবার) গবেষণায় অন্তর্ভুক্ত করেছিলাম। ৬৪০টি (৩৫.৫%) খানাকে ধূমপানমুক্ত বাড়ির জন্য ইন্টারভেনশন এবং গৃহাভ্যন্তরীন বাতাসের গুনগত মানের ফলাফল প্রদান করার দলটিতে অন্তর্ভুক্ত করা হয়েছিল। ৫৬০টি (৩১.১%) খানাকে কেবলমাত্র ধূমপানমুক্ত বাড়ির জন্য ইন্টারভেনশন প্রদান করার দলটিতে অন্তর্ভুক্ত করা হয়েছিল। এবং অবশিষ্ট ৬০১টি (৩৩.৪%) গৃহকে গতানুগতিক সেবা (usual services)-এর দলটিতে অন্তর্ভুক্ত করা হয়েছিল।

প্রতি ২৪ ঘন্টায় গৃহাভ্যন্তরীন বাতাসে উপস্থিত সূক্ষ্ম কণা (যার ব্যাস ২.৫ মাইক্রনের চেয়ে কম অর্থাৎ বস্তুকণা বা পার্টিকুলেট ম্যাটার ২.৫) –এর গড় ঘনত্বের পরিমাণের সমন্বিত গড় পার্থক্য নির্ণয় করার পর ১২তম মাসে যে ফলাফলগুলো পাওয়া গেছে, তা নিম্নরূপঃ

যেসকল খানাতে 'ধূমপানমুক্ত বাড়ি' নামক ইন্টারভেনশন এবং গৃহাভ্যন্তরীন বাতাসের গুণগত মান সংক্রান্ত ফলাফল প্রদান করা হয়েছে; তাদের সাথে তুলনা করলে যে সকল খানাতে 'গতানুগতিক সেবা' প্রদান করা হয়েছে – তাদের গৃহাভ্যন্তরীন বাতাসে উপস্থিত সূক্ষ্ম কণার ঘনত্বের সমন্বিত গড় পার্থক্য ছিল -১.০ মাইক্রোগ্রাম/ঘনমিটার (৯৫% কনফিডেন্স ইন্টারভ্যাল এর মান -১২.৮ থেকে ১০.৯; p-এর মান ০.৮৮)।

যেসকল খানাতে কেবলমাত্র 'ধূমপানমুক্ত বাড়ি' নামক ইন্টারভেনশন প্রদান করা হয়েছে; তাদের সাথে তুলনা করলে যেসকল খানাতে 'গতানুগতিক সেবা' প্রদান করা হয়েছে – তাদের গৃহাভ্যন্তরীন বাতাসে উপস্থিত সূক্ষ্ম কনার ঘনত্বের সমন্বিত গড় পার্থক্য ছিল ৫.০ মাইক্রোগ্রাম/ঘনমিটার (৯৫% কনফিডেন্স ইন্টারভ্যাল –এর মান -৭.৯ থেকে ১৮.০; p-এর মান ০.৪৫)।

যেসকল খানাতে 'ধূমপানমুক্ত বাড়ি' নামক ইন্টারভেনশন এবং গৃহাভ্যন্তরীন বাতাসের গুণগত মান সংক্রান্ত ফলাফল প্রদান করা হয়েছে; তাদের সাথে তুলনা করলে যেসকল খানাতে কেবলমাত্র 'ধূমপানমুক্ত বাড়ি' নামক ইন্টারভেনশন প্রদান করা হয়েছে – তাদের গৃহাভ্যন্তরীন বাতাসে উপস্থিত সূক্ষ্ম কনার ঘনত্বের সমন্বিত গড় পার্থক্য ছিল -৬.০ মাইক্রোগ্রাম/ঘনমিটার (৯৫% কনফিডেন্স ইন্টারভ্যাল –এর মান -১৮.৩ থেকে ৬.৩; p-এর মান ০.৪৫)।

ক্রমবর্ধমান কার্যকারিতা সংক্রান্ত ব্যায় অনুপাত (incremental cost effectiveness ratio) নির্নয়ের ক্ষেত্রে প্রাপ্ত ফলাফল ছিল নিম্নরূপঃ

যেসকল খানাতে 'ধূমপানমুক্ত বাড়ি' নামক ইন্টারভেনশন এবং গৃহাভ্যন্তরীন বাতাসের গুণগত মান সংক্রান্ত ফলাফল প্রদান করা হয়েছে; তাদের সাথে তুলনা করলে যেসকল খানাতে 'গতানুগতিক সেবা' প্রদান করা হয়েছে – সেসকল ক্ষেত্রে জীবনমান অনুসারে মানুষের আয়ু (কোয়ালিটি অ্যাডজাস্টেড লাইফ ইয়ার বা QALY)-এর ক্ষেত্রে বছর-প্রতি এই অনুপাতটি হচ্ছে ৬৫৩ মার্কিন ডলার। এই পরিমাণটি জীবনমান অনুসারে মানুষের আয়ু (কোয়ালিটি অ্যাডজাস্টেড লাইফ ইয়ার বা QALY)-এর ক্ষেত্রে বছর-প্রতি বাংলাদেশীরা যত অর্থ ব্যয় করতে ইচ্ছুক (৪২৭ মার্কিন ডলার), তার চেয়েও বেশি।

'ধূমপানমুক্ত বাড়ি' নামক ইন্টারভেনশন (গৃহাভ্যন্তরীন বাতাসের গুণগত মান সংক্রান্ত ফলাফলসহ কিংবা এই ফলাফল ব্যাতিরেকে) প্রদান করার সাথে গতানুগতিক সেবা (usual services)-র তুলনা করে দেখা গেছে যে, ইন্টারভেনশনটি গৃহাভ্যন্তরে পরোক্ষ ধূমপানের প্রকোপ কমাতে তুলনামূলকভাবে কার্যকর বলে প্রমাণিত হয় নি, কিংবা এর কার্যকারিতা সংক্রান্ত ব্যায় (cost effectiveness) এর ফলপ্রসূতাও ছিল না।

এ কারনে এই ধরনের ইন্টারভেনশন কার্যক্রম বাংলাদেশের জন্য সুপারিশ / প্রস্তাব করা হচ্ছে না।

অর্থায়নেঃ মেডিকেল রিসার্চ কাউন্সিল, যুক্তরাজ্য।

সর্বস্বত্ব সংরক্ষিতঃ ২০২১, সকল লেখকবৃন্দ। এলসেভিয়ার লিঃ দ্বারা প্রকাশিত। এটি CC BY 4.0 লাইসেন্সের আওতাভুক্ত একটি উন্মুক্ত নিবন্ধ।
