# Supplementary material for: Efficacy and cost-effectiveness of a community-based smoke-free-home intervention with or without indoor-air-quality feedback in Bangladesh (MCLASS II): a three-arm, cluster-randomised, controlled trial
Source: Lancet Glob Health. 2021 Apr 15;9(5):e639–50. doi: 10.1016/S2214-109X(21)00040-1 (PMC8064237; doi:10.1016/S2214-109X(21)00040-1)

THE LANCET
Global Health

Supplementary appendix 2

This appendix formed part of the original submission and has been peer reviewed. We post it as supplied by the authors.

Supplement to: Mdege ND, Fairhurst C, Wang H-I, et al. Efficacy and cost-effectiveness of a community-based smoke-free-home intervention with or without indoor-air-quality feedback in Bangladesh (MCLASS II): a three-arm, cluster-randomised, controlled trial. *Lancet Glob Health* 2021; **9:** e639–50.

Title: Efficacy and cost-effectiveness of a community-based smoke-free-home intervention with or without indoor air quality feedback in Bangladesh (MCLASS II): a three-arm, cluster-randomised, controlled trial.

APPENDIX 2

Supplementary material: statistical analysis methods

S1: Primary analysis model assumptions
Visual inspection of model assumptions for the primary analysis, using a QQ-plot for the normality of the standardised residuals and a scatter plot of the standardised residuals against fitted values to assess scedasticity, demonstrated substantial deviations. Log-transformation of the outcome data improved model fit in a sensitivity analysis.

S2: Sensitivity analyses
A total of 75 Dylos devices were calibrated in November 2017 and used during the baseline and three-month follow-up assessments. The 12-month assessments were due to take place between May and August 2019. Dr Sean Semple visited Dhaka in February 2019 to clean the devices, replace the clock batteries and check the performance of the devices against a 'gold-standard' instrument (a Sidepak AM510 Personal Aerosol Monitor) that had recently been factory calibrated, under the assumption that the Dylos devices would measure broadly the same as the Sidepak as was observed in the initial calibration. However, data from the 12-month calibrations showed a much higher variation in Dylos response and a substantial underestimation of $PM_{2·5}$ concentrations relative to the Sidepak device. On average, the Dylos machines underestimated the response by a factor of 2·48.

Dr Sean Semple postulated that the response from the laser particle counter in the Dylos devices had experienced degradation and was not 'seeing' the same particle flow as when the devices was initially calibrated. This laser degradation has been previously alluded to by the developer of the Dylos instrument but we are not aware of any literature describing this phenomenon in air quality measurement literature. The devices had been heavily used during the baseline and three-month follow-up work in 2018 with each device running for approximately (50 x 24) 1200 hours at high concentrations of second-hand smoke (SHS) and ambient air pollution and at the high ambient temperatures experienced in Bangladesh. It seems likely that these exposures resulted in the degradation of the performance of the laser.
The solution proposed was to apply a device-specific 'correction' factor to the responses at the 12-month follow-up to account for the degradation. Application of a correction factor showed a high level of agreement with the Sidepak concentration across the measurement range.

The primary analysis model included baseline and three-month data as originally recorded by the Dylos machine, but 12-month data *had* the correction factor applied. Two sensitivity analyses were conducted to assess the impact of removing/applying the correction factor:
1) Including 'uncorrected' (original) 12-month response data. We back-transformed the 12-month Dylos response data for each device to remove the correction factor, and included these data in the analysis model.
2) We retained the 'corrected' 12-month data and also applied a device-specific correction factor to the three-month data. In theory, if the degradation observed in the second calibration was caused by, and is proportional to, the heavy use at baseline and three months, then the machines would have experienced approximately half the degradation after baseline prior to their use at the three-month time point. Therefore, we applied half the device-specific correction factor to the three-month Dylos response data and used these data in the analysis.

A further, post-hoc sensitivity analysis was conducted adjusting additionally for household-level confounders prespecified in the protocol (number of residents, presence of outdoor space, number of shops that sell cigarettes or tobacco in neighbourhood, and use of kerosene for cooking).

Supplementary Tables

Table S1: Examples of smoke-free-home (SFH) intervention messages and supporting ayahs

Ayah	Health message
Surah Al-Maaida (5), Ayah 4<br>They ask you, [O Muhammad], what has been made lawful for them. Say, "Lawful for you are [all] good foods."	Islam has specific and fundamental sets of rules about drinks and foods. Things that are beneficial for the human body and health, are allowed in Islam and those that are harmful are prohibited for them. Hence, would Allah permit you to consume something harmful? Certainly not!<br><br>Tobacco and products made of tobacco are very harmful and that is why it is not acceptable to Allah. Muslims should lead a lifestyle that is recommended by Allah and His Messenger knowing what is permitted and what is prohibited by them.
Surah At-Baqara – 195 (2:195)<br>And do good; indeed, Allah loves the doers of good.	Those who smoke around us, unintentionally harm others directly. Thus, every year six hundred thousand people die due to exposure to passive smoking worldwide. So, we have to be aware of passive smoking and be careful about smoking inside home and in front of others. We also need to share these messages with others. We must keep ourselves and our families safe from the harm of passive smoking. Allah also loves those who do good things.

Table S2: Follow-up and data collection schedule

Data collected	Baseline	3-month follow-up	6-month follow-up	12-month follow-up
**Socio-demographic variables**	X			
**Self-reported smoking behaviour and restrictions**	X	X	X	X
**Concentration of $PM_{2·5}$ in the home**	X	X		X
**Respiratory symptom**	X	X	X	X
**Health service use**	X	X	X	X
**Health-related quality of life**	X	X	X	X
**Mediators of intervention effectiveness**	X	X	X	X
**Process evaluation data**		X		
**Mosque attendance**	X			
**Mosque attendance and receipt of SFH programme**		X	X	X

Table S3: Reasons for household ineligibility (not mutually exclusive)

Reason	Frequency (% of 4430)
No adult resident who smokes tobacco regularly (at least 25 out of 30 days/month)	1875 (42·3)
Unwilling to give written informed consent	474 (10·7)
Planning to move home in the next 12 months	441 (10·0)
Unable to give written informed consent	296 (6·7)
Do not attend a participating mosque	85 (1·9)
Household uses coal or biomass fuel	84 (1·9)
No member of the household attends a mosque for their regular and/or Friday prayers	72 (1·6)
No resident non-smoker	19 (0·4)

Table S4: Characteristics of randomised mosques (n=45)

	Usual services (n=15)	SFH intervention (n=14)	SFH intervention plus IAQ feedback (n=16)	Total (n=45)
**Area (Ward No.), n (%)**				
02	1 (7·1)	2 (12·5)	2 (13·3)	5 (11·1)
03	1 (7·1)	0 (0·0)	0 (0·0)	1 (2·2)
04	1 (7·1)	1 (6·3)	0 (0·0)	2 (4·4)
05	1 (7·1)	2 (12·5)	2 (13·3)	5 (11·1)
06	1 (7·1)	1 (6·3)	1 (6·7)	3 (6·7)
07	1 (7·1)	1 (6·3)	0 (0·0)	2 (4·4)
08	1 (7·1)	2 (12·5)	2 (13·3)	5 (11·1)
09	1 (7·1)	1 (6·3)	0 (0·0)	2 (4·4)
10	1 (7·1)	1 (6·3)	2 (13·3)	4 (8·9)
11	1 (7·1)	1 (6·3)	1 (6·7)	3 (6·7)
12	1 (7·1)	0 (0·0)	0 (0·0)	1 (2·2)
13	1 (7·1)	1 (6·3)	1 (6·7)	3 (6·7)
14	0 (0·0)	0 (0·0)	1 (6·7)	1 (2·2)
15	1 (7·1)	2 (12·5)	2 (13·3)	5 (11·1)
16	1 (7·1)	1 (6·3)	1 (6·7)	3 (6·7)
**Average estimate size of Friday congregation**				
Mean (SD)	2213·6 (2971·8)	1475·0 (1209·7)	1920·0 (1668·7)	1853·1 (2022·2)
Median (min, max)	1325 (140, 12000)	1100 (200, 5000)	1200 (200, 5000)	1200 (140, 12000)
<=1500, n (%)	9 (64·3)	10 (62·5)	9 (60·0)	28 (62·2)
>1500, n (%)	5 (35·7)	6 (37·5)	6 (40·0)	17 (37·8)
**Average estimates of people who attend two or more daily prayers**				
Mean (SD)	419·3 (628·8)	228·1 (140·0)	315·3 (282·2)	316·7 (393·7)
Median (min, max)	250 (40, 2500)	250 (25, 500)	300 (25, 1000)	250 (25, 2500)
**Average estimate size of male study circle**				
Mean (SD)	58·6 (102·8)	38·4 (48·7)	38·8 (63·3)	44·8 (72·7)
Median (min, max)	30 (10, 400)	17·5 (0, 150)	15 (0, 250)	20 (0, 400)
**Average estimate size of female study circle**[a]				
Mean (SD)	1·2 (4·5)	6·3 (20·3)	0·0 (0·0)	2·6 (12·4)
Median (min, max)	0 (0, 17)	0 (0, 80)	0 (0, 0)	0 (0, 80)
**Average estimate size of Qur'an class**				
Mean (SD)	64·6 (64·4)	71·9 (47·3)	68·0 (48·7)	68·3 (52·4)
Median (min, max)	35 (15, 250)	55 (15, 150)	50 (0, 200)	50 (0, 250)
**Average age (self-reported by teacher) of students/children taught**				
Mean (SD)	8·3 (2·3)	8·7 (1·5)	8·1 (1·7)	8·4 (1·8)
Median (min, max)	8 (6, 15)	9 (7, 12)	8 (4, 11)	8 (4, 15)

[a] only three mosques held female study circles so value of 0 for 42 of the 45 mosques

Table S5: Characteristics of household at baseline (as randomised)

	Usual services (n=601)	SFH intervention (n=560)	SFH intervention plus IAQ feedback (n=640)	Total (n=1801)
**Home has outside space, n (%)**	327 (54·4)	329 (58·8)	316 (49·4)	972 (54·0)
**Number of bedrooms**				
Mean (SD)	1·4 (0·7)	1·5 (0·8)	1·3 (0·6)	1·4 (0·7)
Median (min, max)	1·0 (0·0, 5·0)	1·0 (1·0, 6·0)	1·0 (1·0, 4·0)	1·0 (0·0, 6·0)
**Number of adult residents**				
Mean (SD)	2·4 (0·8)	2·4 (0·8)	2·3 (0·7)	2·4 (0·8)
Median (min, max)	2·0 (1·0, 6·0)	2·0 (1·0, 6·0)	2·0 (1·0, 5·0)	2·0 (1·0, 6·0)
**Number of child residents**				
Mean (SD)	1·3 (1·1)	1·3 (1·1)	1·5 (1·1)	1·4 (1·1)
Median (min, max)	1·0 (0·0, 7·0)	1·0 (0·0, 5·0)	1·0 (0·0, 6·0)	1·0 (0·0, 7·0)
**Total number of residents**				
Mean (SD)	3·8 (1·3)	3·8 (1·3)	3·8 (1·3)	3·8 (1·3)
Median (min, max)	4·0 (2·0, 10·0)	4·0 (2·0, 10·0)	4·0 (2·0, 9·0)	4·0 (2·0, 10·0)
**Household has, n (%)**				
Electricity	601 (100·0)	560 (100·0)	640 (100·0)	1801 (100·0)
Flush toilet	99 (16·5)	48 (8·6)	29 (4·5)	176 (9·8)
Fixed telephone	4 (0·7)	9 (1·6)	5 (0·8)	18 (1·0)
Cell telephone	592 (98·5)	544 (97·1)	624 (97·5)	1760 (97·7)
Television	442 (73·5)	457 (81·6)	491 (76·7)	1390 (77·2)
Radio	14 (2·3)	16 (2·9)	18 (2·8)	48 (2·7)
Refrigerator	287 (47·8)	294 (52·5)	241 (37·7)	822 (45·6)
Car	8 (1·3)	8 (1·4)	6 (0·9)	22 (1·2)
Moped/Scooter/Motorcycle	18 (3·0)	15 (2·7)	12 (1·9)	45 (2·5)
**Type of fuel used for cooking, n (%)**				
Electricity	50 (8·3)	92 (16·4)	13 (2·0)	155 (8·6)
LPG/natural gas/biogas	538 (89·5)	479 (85·5)	637 (99·5)	1654 (91·8)
Kerosene	34 (5·7)	7 (1·3)	23 (3·6)	64 (3·6)
**Home has, n (%)**				
Water leading to structural damage	101 (16·8)	130 (23·2)	118 (18·4)	349 (19·4)
Damp stains or paint peeling but no structural damage	141 (23·5)	110 (19·6)	94 (14·7)	345 (19·2)
Visible mould but no damp stains or paint peeling	120 (20·0)	88 (15·7)	72 (11·3)	280 (15·5)
Mould odour but no visible mould	114 (19·0)	68 (12·1)	99 (15·5)	281 (15·6)
**Household own any cattle (bulls, buffaloes or cows)/pets/poultry (chicken or ducks), n (%)**	26 (4·3)	26 (4·6)	23 (3·6)	75 (4·2)

Table S6: Smoking status of household and lead adult at baseline (as randomised)

	Usual services (n=601)	SFH intervention (n=560)	SFH intervention plus IAQ feedback (n=640)	Total (n=1801)
**Number of adult resident smokers**				
Mean (SD)	1·1 (0·3)	1·1 (0·3)	1·1 (0·3)	1·1 (0·3)
Median (min, max)	1·0 (0·0, 3·0)	1·0 (0·0, 3·0)	1·0 (0·0, 3·0)	1·0 (0·0, 3·0)
**Number of child resident smokers**				
Mean (SD)	0·0 (0·1)	0·0 (0·1)	0·0 (0·1)	0·0 (0·1)
Median (min, max)	0·0 (0·0, 1·0)	0·0 (0·0, 1·0)	0·0 (0·0, 1·0)	0·0 (0·0, 1·0)
**Total number of resident smokers**				
Mean (SD)	1·1 (0·3)	1·1 (0·3)	1·1 (0·3)	1·1 (0·3)
Median (min, max)	1·0 (1·0, 3·0)	1·0 (1·0, 3·0)	1·0 (1·0, 3·0)	1·0 (1·0, 3·0)
**Residents allowed to smoke, n (%)**				
Anywhere inside your home	276 (45·9)	242 (43·2)	353 (55·2)	871 (48·4)
Only in some rooms in your home	5 (0·8)	7 (1·3)	2 (0·3)	14 (0·8)
Only in one room in your home	19 (3·2)	43 (7·7)	22 (3·4)	84 (4·7)
Only outside	298 (49·6)	265 (47·3)	260 (40·6)	823 (45·7)
Don't know	3 (0·5)	3 (0·5)	3 (0·5)	9 (0·5)
**Visitors are allowed to smoke, n (%)**				
Anywhere inside your home	246 (40·9)	201 (35·9)	334 (52·2)	781 (43·4)
Only in some rooms in your home	4 (0·7)	6 (1·1)	3 (0·5)	13 (0·7)
Only in one room in your home	15 (2·5)	34 (6·1)	17 (2·7)	66 (3·7)
Only outside	324 (53·9)	309 (55·2)	276 (43·1)	909 (50·5)
Don't know	12 (2·0)	10 (1·8)	10 (1·6)	32 (1·8)
**Are residents allowed to smoke in front of children in the home?, n (%)**				
Yes	185 (30·8)	187 (33·4)	259 (40·5)	631 (35·0)
No	294 (48·9)	245 (43·8)	254 (39·7)	793 (44·0)
Don't know	7 (1·2)	5 (0·9)	12 (1·9)	24 (1·3)
No children live in this house	115 (19·1)	123 (22·0)	115 (18·0)	353 (19·6)
**Are visitors allowed to smoke in front of children in the home?, n (%)**				
Yes	165 (27·5)	154 (27·5)	259 (40·5)	578 (32·1)
No	309 (51·4)	269 (48·0)	251 (39·2)	829 (46·0)
Don't know	15 (2·5)	15 (2·7)	15 (2·3)	45 (2·5)
No children live in this house	112 (18·6)	122 (21·8)	115 (18·0)	349 (19·4)

Table S7: Characteristics of household lead adult at baseline (as randomised)

	Usual services (n=601)	SFH intervention (n=560)	SFH intervention plus IAQ feedback (n=640)	Total (n=1801)
**Age, years**				
Mean (SD)	40·7 (12·5)	40·6 (12·8)	39·9 (12·2)	40·4 (12·5)
Median (min, max)	38·2 (20·1, 85·4)	37·0 (22·1, 85·5)	37·0 (18·4, 84·7)	37·7 (18·4, 85·5)
**Gender (male), n (%)**	572 (95·2)	524 (93·6)	618 (96·6)	1714 (95·2)
**Highest level of education attained, n (%)**				
No education	91 (15·1)	114 (20·4)	151 (23·6)	356 (19·8)
Primary Incomplete	127 (21·1)	117 (20·9)	129 (20·2)	373 (20·7)
Primary Complete	87 (14·5)	71 (12·7)	91 (14·2)	249 (13·8)
Secondary Incomplete	100 (16·6)	92 (16·4)	123 (19·2)	315 (17·5)
Secondary Complete	53 (8·8)	41 (7·3)	31 (4·8)	125 (6·9)
High school Incomplete	6 (1·0)	6 (1·1)	3 (0·5)	15 (0·8)
High school Complete	37 (6·2)	37 (6·6)	24 (3·8)	98 (5·4)
University/ College Incomplete	10 (1·7)	0 (0·0)	2 (0·3)	12 (0·7)
University/ College Complete	26 (4·3)	47 (8·4)	32 (5·0)	105 (5·8)
Other	64 (10·6)	35 (6·3)	54 (8·4)	153 (8·5)
**Presently smoke, n (%)**				
Not at all	60 (10·0)	78 (13·9)	44 (6·9)	182 (10·1)
Sometimes	17 (2·8)	20 (3·6)	17 (2·7)	54 (3·0)
Everyday	524 (87·2)	462 (82·5)	579 (90·5)	1565 (86·9)
**If yes[a]:**				
**Form of tobacco, n (%)**				
Cigarettes	529 (97·8)	474 (98·3)	584 (98·0)	1587 (98·0)
Per day, mean (SD)	12·7 (7·0)	12·0 (6·6)	12·0 (7·4)	12·2 (7·0)
Bidi	32 (5·9)	10 (2·1)	37 (6·2)	79 (4·9)
Per day, mean (SD)	14·5 (7·5)	9·7 (6·6)	14·6 (8·0)	13·9 (7·7)
**Number of days smoked in last 30 days**				
Mean (SD)	29·7 (1·0)	29·8 (1·1)	29·9 (0·7)	29·8 (1·0)
Median (min, max)	30·0 (25, 30)	30·0 (20, 30)	30·0 (25, 30)	30·0 (20, 30)
**Seriously thinking about quitting, n (%)**				
No	336 (62·1)	224 (46·5)	319 (53·5)	879 (54·3)
Yes, in the next 4 weeks	50 (9·2)	65 (13·5)	38 (6·4)	153 (9·5)
Yes, in the next 6 months	155 (28·7)	193 (40·0)	239 (40·1)	587 (36·3)
**Seriously tried quitting smoking, n (%)**				
Yes	115 (21·3)	145 (30·1)	222 (37·2)	482 (29·8)
No	426 (78·7)	337 (69·9)	374 (62·8)	1137 (70·2)
**If no[b], ever smoked in the past, n (%)**				
Never smoked	55 (91·7)	72 (92·3)	40 (90·9)	167 (91·8)
Yes, sometimes	2 (3·3)	1 (1·3)	3 (6·8)	6 (3·3)
Yes, regularly	3 (5·0)	5 (6·4)	1 (2·3)	9 (4·9)

[a] Currently smoke sometimes or everyday

[b] Do not currently smoke

Table S8: Household mosque attendance at baseline (as randomised)

	Usual services (n=601)	SFH intervention (n=560)	SFH intervention plus IAQ feedback (n=640)	Total (n=1801)
**HH lead regularly attends a mosque for your daily and/or Friday prayers, n (%)**				
Yes	571 (95·0)	525 (93·8)	621 (97·0)	1717 (95·3)
No	30 (5·0)	35 (6·3)	19 (3·0)	84 (4·7)
**If yes:**				
**Attend, n (%)**				
Qu'ran Classes	0 (0·0)	0 (0·0)	3 (0·5)	3 (0·2)
Regular prayers (excluding Friday Jumu'ah Prayers)	102 (17·9)	103 (19·6)	91 (14·7)	296 (17·2)
Friday Jumu'ah Prayers	563 (98·6)	517 (98·5)	616 (99·2)	1696 (98·8)
Study Circle	36 (6·3)	29 (5·5)	8 (1·3)	73 (4·3)
Other[a]	14 (2·5)	5 (1·0)	5 (0·8)	24 (1·4)
**Attend more than one mosque for your daily and/or Friday prayers, n (%)**				
Yes	23 (4·0)	111 (21·1)	75 (12·1)	209 (12·2)
No	548 (96·0)	414 (78·9)	546 (87·9)	1508 (87·8)
**Regularity of attendance at mosque, n (%)**				
Daily	99 (17·3)	99 (18·9)	86 (13·8)	284 (16·5)
Weekly	472 (82·7)	423 (80·6)	535 (86·2)	1430 (83·3)
Monthly	0 (0·0)	2 (0·4)	0 (0·0)	2 (0·1)
Prefer not to say	0 (0·0)	1 (0·2)	0 (0·0)	1 (0·1)
**Other members of household attend a mosque, n (%)**				
Yes	258 (42·9)	269 (48·0)	259 (40·5)	786 (43·6)
No	343 (57·1)	291 (52·0)	381 (59·5)	1015 (56·4)
**If yes:**				
**Attend, n (%)**				
Qu'ran Classes for Adults	3 (1·2)	1 (0·4)	1 (0·4)	5 (0·6)
Qu'ran Classes for Children	45 (17·4)	37 (13·8)	48 (18·5)	130 (16·5)
Regular prayers (excluding Friday Jumu'ah Prayers)	55 (21·3)	50 (18·6)	52 (20·1)	157 (20·0)
Friday Jumu'ah Prayers	234 (90·7)	245 (91·1)	239 (92·3)	718 (91·3)
Study Circle	33 (12·8)	25 (9·3)	7 (2·7)	65 (8·3)
Don't know	0 (0·0)	1 (0·4)	0 (0·0)	1 (0·1)
Other[b]	4 (1·6)	1 (0·4)	1 (0·4)	6 (0·8)

[a] all irregular weekly mosque attendance

[b] Control: irregular weekly mosque attendance (n=4); M4BH alone: irregular weekly mosque attendance (n=1); M4BH and IAQ: milad (n=1)

Table S9: Characteristics of household at baseline (as analysed)

	Usual services (n=450)	SFH intervention (n=420)	SFH intervention plus IAQ feedback (n=480)	Total (n=1350)
**Total number of residents**				
Mean (SD)	3·8 (1·4)	3·8 (1·3)	3·8 (1·3)	3·8 (1·3)
Median (min, max)	4·0 (2·0, 10·0)	4·0 (2·0, 10·0)	4·0 (2·0, 9·0)	4·0 (2·0, 10·0)
**Household has, n (%)**				
Electricity	450 (100·0)	420 (100·0)	480 (100·0)	1350 (100·0)
Flush toilet	75 (16·7)	31 (7·4)	20 (4·2)	126 (9·3)
Fixed telephone	4 (0·9)	6 (1·4)	2 (0·4)	12 (0·9)
Cell telephone	447 (99·3)	410 (97·6)	467 (97·3)	1324 (98·1)
Television	340 (75·6)	344 (81·9)	372 (77·5)	1056 (78·2)
Radio	14 (3·1)	10 (2·4)	14 (2·9)	38 (2·8)
Refrigerator	217 (48·2)	219 (52·1)	187 (39·0)	623 (46·1)
Car	7 (1·6)	6 (1·4)	2 (0·4)	15 (1·1)
Moped/Scooter/Motorcycle	15 (3·3)	11 (2·6)	7 (1·5)	33 (2·4)
**Home has, n (%)**				
Water leading to structural damage	73 (16·2)	96 (22·9)	87 (18·1)	256 (19·0)
Damp stains or paint peeling but no structural damage	99 (22·0)	85 (20·2)	68 (14·2)	252 (18·7)
Visible mould but no damp stains or paint peeling	81 (18·0)	67 (16·0)	52 (10·8)	200 (14·8)
Mould odour but no visible mould	84 (18·7)	56 (13·3)	81 (16·9)	221 (16·4)
**Household own any cattle (bulls, buffaloes or cows)/pets/poultry (chicken or ducks), n (%)**	22 (4·9)	23 (5·5)	17 (3·5)	62 (4·6)

Table S10: Smoking status of household and lead adult at baseline (as analysed)

	Usual services (n=450)	SFH intervention (n=420)	SFH intervention plus IAQ feedback (n=480)	Total (n=1350)
**Total number of resident smokers**				
Mean (SD)	1·1 (0·3)	1·1 (0·3)	1·1 (0·3)	1·1 (0·3)
Median (min, max)	1·0 (1·0, 3·0)	1·0 (1·0, 3·0)	1·0 (1·0, 3·0)	1·0 (1·0, 3·0)
**Residents allowed to smoke, n (%)**				
Anywhere inside your home	197 (43·8)	180 (42·9)	262 (54·6)	639 (47·3)
Only in some rooms in your home	5 (1·1)	6 (1·4)	1 (0·2)	12 (0·9)
Only in one room in your home	14 (3·1)	36 (8·6)	15 (3·1)	65 (4·8)
Only outside	233 (51·8)	196 (46·7)	199 (41·5)	628 (46·5)
Don’t know	1 (0·2)	2 (0·5)	3 (0·6)	6 (0·4)
**Visitors are allowed to smoke, n (%)**				
Anywhere inside your home	178 (39·6)	146 (34·8)	247 (51·5)	571 (42·3)
Only in some rooms in your home	4 (0·9)	6 (1·4)	1 (0·2)	11 (0·8)
Only in one room in your home	11 (2·4)	30 (7·1)	12 (2·5)	53 (3·9)
Only outside	250 (55·6)	229 (54·5)	213 (44·4)	692 (51·3)
Don’t know	7 (1·6)	9 (2·1)	7 (1·5)	23 (1·7)
**Are residents allowed to smoke in front of children in the home?, n (%)**				
Yes	132 (29·3)	142 (33·8)	194 (40·4)	468 (34·7)
No	221 (49·1)	179 (42·6)	191 (39·8)	591 (43·8)
Don’t know	6 (1·3)	3 (0·7)	10 (2·1)	19 (1·4)
No children live in this house	91 (20·2)	96 (22·9)	85 (17·7)	272 (20·1)
**Are visitors allowed to smoke in front of children in the home?, n (%)**				
Yes	118 (26·2)	112 (26·7)	196 (40·8)	426 (31·6)
No	234 (52·0)	201 (47·9)	185 (38·5)	620 (45·9)
Don’t know	9 (2·0)	12 (2·9)	14 (2·9)	35 (2·6)
No children live in this house	89 (19·8)	95 (22·6)	85 (17·7)	269 (19·9)

Table S11: Characteristics of household lead adult at baseline (as analysed)

	Usual services (n=450)	SFH intervention (n=420)	SFH intervention plus IAQ feedback (n=480)	Total (n=1350)
**Age, years**				
Mean (SD)	40·8 (12·8)	40·7 (12·7)	40·3 (12·1)	40·6 (12·5)
Median (min, max)	38·2 (20·1, 85·4)	37·3 (22·1, 85·5)	38·1 (20·2, 84·7)	38·1 (20·1, 85·5)
**Gender (male), n (%)**	429 (95·3)	387 (92·1)	461 (96·0)	1277 (94·6)
**Highest level of education attained, n (%)**				
No education	65 (14·4)	90 (21·4)	113 (23·5)	268 (19·9)
Primary Incomplete	101 (22·4)	82 (19·5)	86 (17·9)	269 (19·9)
Primary Complete	68 (15·1)	57 (13·6)	79 (16·5)	204 (15·1)
Secondary Incomplete	77 (17·1)	74 (17·6)	96 (20·0)	247 (18·3)
Secondary Complete	40 (8·9)	29 (6·9)	21 (4·4)	90 (6·7)
High school Incomplete	4 (0·9)	3 (0·7)	2 (0·4)	9 (0·7)
High school Complete	21 (4·7)	25 (6·0)	16 (3·3)	62 (4·6)
University/ College Incomplete	7 (1·6)	0 (0·0)	2 (0·4)	9 (0·7)
University/ College Complete	19 (4·2)	35 (8·3)	25 (5·2)	79 (5·9)
Other	48 (10·7)	25 (6·0)	40 (8·3)	113 (8·4)
**Presently smoke, n (%)**				
Not at all	44 (9·8)	61 (14·5)	40 (8·3)	145 (10·7)
Sometimes	12 (2·7)	14 (3·3)	13 (2·7)	39 (2·9)
Everyday	394 (87·6)	345 (82·1)	427 (89·0)	1166 (86·4)
**If yes[a]:**				
**Form of tobacco, n (%)**				
Cigarettes	395 (97·3)	355 (98·9)	429 (97·5)	1179 (97·8)
Per day, mean (SD)	12·4 (6·7)	12·0 (6·6)	12·3 (7·8)	12·2 (7·1)
Bidi	24 (5·9)	5 (1·4)	27 (6·1)	56 (4·6)
Per day, mean (SD)	16·2 (7·6)	10·6 (8·9)	13·9 (7·3)	14·6 (7·6)
**Number of days smoked in last 30 days**				
Mean (SD)	29·8 (1·0)	29·8 (1·1)	29·9 (0·7)	29·8 (1·0)
Median (min, max)	30 (25, 30)	30 (20, 30)	30 (25, 30)	30 (20, 30)
**Seriously thinking about quitting, n (%)**				
No	243 (59·9)	164 (45·7)	232 (52·7)	639 (53·0)
Yes, in the next 4 weeks	37 (9·1)	47 (13·1)	25 (5·7)	109 (9·0)
Yes, in the next 6 months	126 (31·0)	148 (41·2)	183 (41·6)	457 (37·9)
**Seriously tried quitting smoking, n (%)**				
Yes	92 (22·7)	110 (30·6)	168 (38·2)	370 (30·7)
No	314 (77·3)	249 (69·4)	272 (61·8)	835 (69·3)
**If no[b], ever smoked in the past, n (%)**				
Never smoked	40 (90·9)	56 (91·8)	36 (90·0)	132 (91·0)
Yes, sometimes	2 (4·5)	1 (1·6)	3 (7·5)	6 (4·1)
Yes, regularly	2 (4·5)	4 (6·6)	1 (2·5)	7 (4·8)

[a] Currently smoke sometimes or everyday

[b] Do not currently smoke

Table S12: Household mosque attendance at baseline (as analysed)

	Usual services (n=450)	SFH intervention (n=420)	SFH intervention plus IAQ feedback (n=480)	Total (n=1350)
**HH lead regularly attends a mosque for your daily and/or Friday prayers, n (%)**				
Yes	429 (95·3)	388 (92·4)	463 (96·5)	1280 (94·8)
No	21 (4·7)	32 (7·6)	17 (3·5)	70 (5·2)
**If yes:**				
**Attend, n (%)**				
Qu'ran Classes	0 (0·0)	0 (0·0)	1 (0·2)	1 (0·1)
Regular prayers (excluding Friday Jumu'ah Prayers)	78 (18·2)	70 (18·0)	75 (16·2)	223 (17·4)
Friday Jumu'ah Prayers	425 (99·1)	380 (97·9)	460 (99·4)	1265 (98·8)
Study Circle	31 (7·2)	18 (4·6)	5 (1·1)	54 (4·2)
Other[a]	12 (2·8)	4 (1·0)	4 (0·9)	20 (1·6)
**Attend more than one mosque for your daily and/or Friday prayers, n (%)**				
Yes	16 (3·7)	78 (20·1)	58 (12·5)	152 (11·9)
No	413 (96·3)	310 (79·9)	405 (87·5)	1128 (88·1)
**Regularity of attendance at mosque, n (%)**				
Daily	76 (17·7)	67 (17·3)	71 (15·3)	214 (16·7)
Weekly	353 (82·3)	319 (82·2)	392 (84·7)	1064 (83·1)
Monthly	0 (0·0)	1 (0·3)	0 (0·0)	1 (0·1)
Prefer not to say	0 (0·0)	1 (0·3)	0 (0·0)	1 (0·1)
**Other members of household attend a mosque, n (%)**				
Yes	190 (42·2)	203 (48·3)	202 (42·1)	595 (44·1)
No	260 (57·8)	217 (51·7)	278 (57·9)	755 (55·9)
**If yes:**				
**Attend, n (%)**				
Qu'ran Classes for Adults	2 (1·1)	1 (0·5)	0 (0·0)	3 (0·5)
Qu'ran Classes for Children	27 (14·2)	29 (14·3)	38 (18·8)	94 (15·8)
Regular prayers (excluding Friday Jumu'ah Prayers)	41 (21·6)	37 (18·2)	43 (21·3)	121 (20·3)
Friday Jumu'ah Prayers	173 (91·1)	185 (91·1)	187 (92·6)	545 (91·6)
Study Circle	25 (13·2)	14 (6·9)	6 (3·0)	45 (7·6)
Don't know	0 (0·0)	1 (0·5)	0 (0·0)	1 (0·2)
Other[b]	3 (1·6)	1 (0·5)	1 (0·5)	5 (0·8)

[a] all irregular weekly mosque attendance

[b] Control: irregular weekly mosque attendance (n=3); M4BH alone: irregular weekly mosque attendance (n=1); M4BH and IAQ: milad (n=1)

Table S13: St George's Respiratory Questionnaire (all adults) by randomised group and time point

SGRQ		Usual services	SFH intervention	SFH intervention plus IAQ feedback	Total
Baseline (as randomised)	N	1461	1370	1484	4315
	Mean (SD)	11·5 (17·2)	12·4 (17·3)	12·1 (18·1)	12·0 (17·5)
	Median (min, max)	2·7 (0·0, 100·0)	2·7 (0·0, 95·2)	2·7 (0·0, 100·0)	2·7 (0·0, 100·0)
Baseline (as analysed)	N	1087	1024	1109	3220
	Mean (SD)	11·7 (17·2)	12·5 (17·8)	12·2 (18·1)	12·2 (17·7)
	Median (min, max)	2·7 (0·0, 100·0)	2·7 (0·0, 95·2)	2·7 (0·0, 100·0)	2·7 (0·0, 100·0)
Month 3	N	1087	1024	1107	3218
	Mean (SD)	9·4 (15·0)	10·1 (14·7)	10·0 (14·6)	9·8 (14·8)
	Median (min, max)	2·7 (0·0, 97·6)	2·7 (0·0, 90·3)	2·7 (0·0, 95·0)	2·7 (0·0, 97·6)
Month 6	N	1083	1013	1084	3180
	Mean (SD)	9·1 (14·0)	11·3 (15·4)	8·7 (12·0)	9·7 (13·9)
	Median (min, max)	2·7 (0·0, 92·8)	2·7 (0·0, 95·0)	2·7 (0·0, 92·7)	2·7 (0·0, 95·0)
Month 12	N	1047	977	1073	3097
	Mean (SD)	7·7 (12·3)	8·3 (12·4)	6·9 (12·3)	7·6 (12·4)
	Median (min, max)	2·7 (0·0, 92·8)	2·7 (0·0, 84·4)	2·7 (0·0, 92·4)	2·7 (0·0, 92·8)

Table S14: St George's Respiratory Questionnaire (11-17 years) by randomised group and time point

SGRQ		Usual services	SFH intervention	SFH intervention plus IAQ feedback	Total
Baseline (as randomised)	N	263	306	329	898
	Mean (SD)	4·4 (10·0)	6·2 (10·7)	5·8 (11·9)	5·5 (11·0)
	Median (min, max)	0·0 (0·0, 81·6)	2·7 (0·0, 77·4)	0·0 (0·0, 71·3)	0·0 (0·0, 81·6)
Baseline (as analysed)	N	191	233	259	683
	Mean (SD)	5·1 (11·3)	6·4 (11·0)	6·1 (12·6)	5·9 (11·7)
	Median (min, max)	0·0 (0·0, 81·6)	2·7 (0·0, 77·4)	0·0 (0·0, 71·3)	0·0 (0·0, 81·6)
Month 3	N	191	233	259	683
	Mean (SD)	4·2 (8·0)	4·7 (8·9)	5·4 (11·4)	4·8 (9·7)
	Median (min, max)	0·0 (0·0, 40·9)	0·0 (0·0, 47·7)	0·0 (0·0, 79·3)	0·0 (0·0, 79·3)
Month 6	N	190	228	249	667
	Mean (SD)	3·5 (7·7)	5·5 (10·7)	5·0 (11·0)	4·8 (10·1)
	Median (min, max)	0·0 (0·0, 33·9)	0·0 (0·0, 67·9)	0·0 (0·0, 100·0)	0·0 (0·0, 100·0)
Month 12	N	187	219	243	649
	Mean (SD)	2·7 (6·2)	2·9 (7·5)	2·9 (6·8)	2·8 (6·9)
	Median (min, max)	0·0 (0·0, 45·4)	0·0 (0·0, 57·2)	0·0 (0·0, 42·3)	0·0 (0·0, 57·2)

Table S15: Respiratory symptoms (<11 years) by randomised group and time point

Respiratory symptoms total		Usual services	SFH intervention	SFH intervention plus IAQ feedback	Total
Baseline (as randomised)	N	538	448	614	1600
	Mean (SD)	6·4 (12·4)	6·9 (11·4)	6·2 (11·1)	6·5 (11·6)
	Median (min, max)	2 (0, 135)	3 (0, 99)	2 (0, 102)	2 (0, 135)
Baseline (as analysed)	N	409	335	456	1200
	Mean (SD)	6·3 (12·4)	7·2 (10·9)	6·2 (11·2)	6·5 (11·6)
	Median (min, max)	2 (0, 135)	3 (0, 78)	1 (0, 102)	2 (0, 135)
Month 3	N	409	335	456	1200
	Mean (SD)	5·3 (8·8)	5·9 (8·8)	4·0 (8·1)	5·0 (8·6)
	Median (min, max)	2 (0, 75)	3 (0, 80)	1 (0, 108)	2 (0, 108)
Month 6	N	408	332	447	1187
	Mean (SD)	4·4 (6·3)	4·9 (6·8)	4·0 (5·8)	4·4 (6·3)
	Median (min, max)	2 (0, 39)	2 (0, 41)	2 (0, 36)	2 (0, 41)
Month 12	N	397	319	437	1153
	Mean (SD)	2·9 (5·3)	3·4 (5·9)	3·1 (5·2)	3·1 (5·4)
	Median (min, max)	0 (0, 44)	1 (0, 72)	0 (0, 36)	0 (0, 72)

Table S16: Respiratory symptoms - all participants (standardised scores) - by randomised group and time point

Respiratory symptoms total		Usual services	SFH intervention	SFH intervention plus IAQ feedback	Total
Baseline (as analysed)	N	1687	1592	1824	5103
	Mean (SD)	-0·0 (1·0)	0·0 (1·0)	-0·0 (1·0)	0·0 (1·0)
	Median (min, max)	-0·5 (-0·7, 11·1)	-0·4 (-0·7, 6·2)	-0·5 (-0·7, 8·3)	-0·5 (-0·7, 11·1)
Month 3	N	1687	1592	1822	5101
	Mean (SD)	-0·0 (1·0)	0·0 (1·0)	-0·0 (1·0)	-0·0 (1·0)
	Median (min, max)	-0·5 (-0·7, 8·2)	-0·5 (-0·7, 8·7)	-0·5 (-0·7, 12·0)	-0·5 (-0·7, 12·0)
Month 6	N	1681	1573	1780	5034
	Mean (SD)	-0·0 (1·0)	0·1 (1·1)	-0·1 (0·9)	-0·0 (1·0)
	Median (min, max)	-0·5 (-0·7, 6·0)	-0·5 (-0·7, 6·3)	-0·5 (-0·7, 9·5)	-0·5 (-0·7, 9·5)
Month 12	N	1631	1515	1753	4899
	Mean (SD)	-0·0 (1·0)	0·0 (1·0)	-0·0 (1·0)	-0·0 (1·0)
	Median (min, max)	-0·4 (-0·6, 7·5)	-0·4 (-0·6, 12·7)	-0·4 (-0·6, 6·9)	-0·4 (-0·6, 12·7)

Table S17: Respiratory symptoms analysis results

Follow-up	Comparison	Adjusted mean difference (95% CI)	p-value
**St George's Respiratory Questionnaire – all adults**			
Month 3	SFH intervention plus IAQ feedback vs. Usual services	0·5 (-1·6 to 2·6)	0·63
	SFH intervention vs. Usual services	0·4 (-1·9 to 2·7)	0·71
	SFH intervention vs. SFH intervention plus IAQ feedback	-0·1 (-2·3 to 2·1)	0·94
Month 6	SFH intervention plus IAQ feedback vs. Usual services	-0·5 (-2·6 to 1·6)	0·63
	SFH intervention vs. Usual services	1·9 (-0·4 to 4·2)	0·10
	SFH intervention vs. SFH intervention plus IAQ feedback	2·4 (0·3 to 4·6)	0·03
Month 12	SFH intervention plus IAQ feedback vs. Usual services	-0·9 (-3·0 to 1·1)	0·37
	SFH intervention vs. Usual services	0·3 (-2·0 to 2·5)	0·81
	SFH intervention vs. SFH intervention plus IAQ feedback	1·2 (-0·9 to 3·4)	0·26
**St George's Respiratory Questionnaire – 11-17 years**			
Month 3	SFH intervention plus IAQ feedback vs. Usual services	1·1 (-1·2 to 3·5)	0·34
	SFH intervention vs. Usual services	0·7 (-1·9 to 3·2)	0·60
	SFH intervention vs. SFH intervention plus IAQ feedback	-0·5 (-2·8 to 1·9)	0·70
Month 6	SFH intervention plus IAQ feedback vs. Usual services	1·5 (-0·9 to 3·9)	0·22
	SFH intervention vs. Usual services	2·3 (-0·3 to 4·8)	0·09
	SFH intervention vs. SFH intervention plus IAQ feedback	0·8 (-1·7 to 3·2)	0·54
Month 12	SFH intervention plus IAQ feedback vs. Usual services	0·2 (-1·9 to 2·2)	0·87
	SFH intervention vs. Usual services	0·5 (-1·7 to 2·6)	0·69
	SFH intervention vs. SFH intervention plus IAQ feedback	0·3 (-1·8 to 2·3)	0·79
**Respiratory Symptoms – <11 years**			
Month 3	SFH intervention plus IAQ feedback vs. Usual services	-1·1 (-2·5 to 0·3)	0·11
	SFH intervention vs. Usual services	0·8 (-0·7 to 2·4)	0·27
	SFH intervention vs. SFH intervention plus IAQ feedback	2·0 (0·5 to 3·4)	0·01
Month 6	SFH intervention plus IAQ feedback vs. Usual services	-0·2 (-1·4 to 0·9)	0·72
	SFH intervention vs. Usual services	0·7 (-0·6 to 1·9)	0·30
	SFH intervention vs. SFH intervention plus IAQ feedback	0·9 (-0·3 to 2·1)	0·15
Month 12	SFH intervention plus IAQ feedback vs. Usual services	0·4 (-0·7 to 1·5)	0·48
	SFH intervention vs. Usual services	0·7 (-0·5 to 1·9)	0·25
	SFH intervention vs. SFH intervention plus IAQ feedback	0·3 (-0·8 to 1·4)	0·60
**Respiratory questionnaires – all participants (standardised scores)**			
Month 3	SFH intervention plus IAQ feedback vs. Usual services	-0·01 (-0·14 to 0·13)	0·92
	SFH intervention vs. Usual services	0·03 (-0·12 to 0·18)	0·72
	SFH intervention vs. SFH intervention plus IAQ feedback	0·03 (-0·11 to 0·17)	0·63
Month 6	SFH intervention plus IAQ feedback vs. Usual services	-0·03 (-0·16 to 0·11)	0·72
	SFH intervention vs. Usual services	0·12 (-0·03 to 0·27)	0·12
	SFH intervention vs. SFH intervention plus IAQ feedback	0·14 (0·00 to 0·29)	0·04
Month 12	SFH intervention plus IAQ feedback vs. Usual services	-0·04 (-0·18 to 0·10)	0·57
	SFH intervention vs. Usual services	0·03 (-0·12 to 0·18)	0·68
	SFH intervention vs. SFH intervention plus IAQ feedback	0·07 (-0·07 to 0·21)	0·32

Table S18: Smoking restrictions in the household at 12 months

	Usual services (n=441)	SFH intervention (n=404)	SFH intervention plus IAQ feedback (n=467)	Total (n=1312)
**Number of adult resident smokers**				
Mean (SD)	1·0 (0·3)	1·0 (0·4)	1·0 (0·4)	1·0 (0·4)
Median (min, max)	1·0 (0·0, 3·0)	1·0 (0·0, 3·0)	1·0 (0·0, 4·0)	1·0 (0·0, 4·0)
**Number of child resident smokers**				
Mean (SD)	0·0 (0·0)	0·0 (0·1)	0·0 (0·1)	0·0 (0·1)
Median (min, max)	0·0 (0·0, 1·0)	0·0 (0·0, 1·0)	0·0 (0·0, 1·0)	0·0 (0·0, 1·0)
**Residents allowed to smoke, n (%)**				
Anywhere inside your home	110 (24·9)	76 (18·8)	115 (24·6)	301 (22·9)
Only in some rooms in your home	1 (0·2)	2 (0·5)	5 (1·1)	8 (0·6)
Only in one room in your home	54 (12·2)	24 (5·9)	22 (4·7)	100 (7·6)
Only outside	274 (62·1)	299 (74·0)	322 (69·0)	895 (68·2)
Don’t know	2 (0·5)	3 (0·7)	3 (0·6)	8 (0·6)
**Visitors are allowed to smoke, n (%)**				
Anywhere inside your home	90 (20·4)	64 (15·8)	109 (23·3)	263 (20·0)
Only in some rooms in your home	4 (0·9)	0 (0·0)	4 (0·9)	8 (0·6)
Only in one room in your home	32 (7·3)	16 (4·0)	14 (3·0)	62 (4·7)
Only outside	305 (69·2)	310 (76·7)	336 (71·9)	951 (72·5)
Don’t know	10 (2·3)	14 (3·5)	4 (0·9)	28 (2·1)
**Are residents allowed to smoke in front of children in the home?, n (%)**				
Yes	296 (67·1)	262 (64·9)	293 (62·7)	851 (64·9)
No	59 (13·4)	47 (11·6)	89 (19·1)	195 (14·9)
Don’t know	3 (0·7)	1 (0·2)	3 (0·6)	7 (0·5)
No children live in this house	83 (18·8)	94 (23·3)	82 (17·6)	259 (19·7)
**Are visitors allowed to smoke in front of children in the home?, n (%)**				
Yes	307 (69·6)	264 (65·3)	297 (63·6)	868 (66·2)
No	45 (10·2)	35 (8·7)	85 (18·2)	165 (12·6)
Don’t know	5 (1·1)	11 (2·7)	3 (0·6)	19 (1·4)
No children live in this house	84 (19·0)	94 (23·3)	82 (17·6)	260 (19·8)

Table S19: Sensitivity $PM_{2\cdot5}$ Dylos measurements

Average $PM_{2\cdot5}$ measurement (ug/m^3)		Usual services	SFH intervention	SFH intervention plus IAQ feedback	Total
Month 3 – corrected	N	450	420	480	1350
	Mean (SD)	100·4 (103·8)	91·7 (110·1)	94·3 (112·5)	95·5 (108·9)
	Median (min, max)	55·8 (1·1, 578·3)	45·7 (3·7, 798·2)	46·6 (1·0, 651·9)	48·3 (1·0, 798·2)
Month 12 – uncorrected	N	441	405	468	1314
	Mean (SD)	26·8 (19·3)	30·2 (23·4)	26·0 (16·1)	27·6 (19·7)
	Median (min, max)	22·3 (4·9, 168·9)	24·9 (5·7, 170·1)	23·1 (5·5, 128·6)	23·4 (4·9, 170·1)

Table S20: Sensitivity $PM_{2·5}$ Dylos results

Follow-up	Comparison	Adjusted mean difference (95% CI)	p-value
**Month three and 12 'uncorrected'**			
Month 3	SFH intervention plus IAQ feedback vs. Usual services	-10·3 (-21·6 to 1·0)	0·07
	SFH intervention vs. Usual services	-9·6 (-21·4 to 2·3)	0·11
	SFH intervention vs. SFH intervention plus IAQ feedback	0·8 (-10·8 to 12·3)	0·90
Month 12	SFH intervention plus IAQ feedback vs. Usual services	-0·3 (-5·6 to 5·0)	0·90
	SFH intervention vs. Usual services	3·9 (-1·9 to 9·7)	0·19
	SFH intervention vs. SFH intervention plus IAQ feedback	4·2 (-1·3 to 9·8)	0·13
Log-transformed			
Month 3	SFH intervention plus IAQ feedback vs. Usual services	-0·11 (-0·28 to 0·06)	0·20
	SFH intervention vs. Usual services	-0·11 (-0·30 to 0·07)	0·23
	SFH intervention vs. SFH intervention plus IAQ feedback	-0·00 (-0·18 to 0·18)	0·99
Month 12	SFH intervention plus IAQ feedback vs. Usual services	0·00 (-0·15 to 0·15)	0·99
	SFH intervention vs. Usual services	0·10 (-0·07 to 0·26)	0·26
	SFH intervention vs. SFH intervention plus IAQ feedback	0·09 (-0·06 to 0·25)	0·24
**Month three and 12 'corrected'**			
Month 3	SFH intervention plus IAQ feedback vs. Usual services	-8·2 (-25·0 to 8·6)	0·34
	SFH intervention vs. Usual services	-8·3 (-26·1 to 9·6)	0·36
	SFH intervention vs. SFH intervention plus IAQ feedback	-0·1 (-17·3 to 17·1)	0·99
Month 12	SFH intervention plus IAQ feedback vs. Usual services	-0·8 (-12·9 to 11·2)	0·89
	SFH intervention vs. Usual services	4·9 (-8·3 to 18·1)	0·46
	SFH intervention vs. SFH intervention plus IAQ feedback	5·7 (-6·7 to 18·2)	0·37
Log-transformed			
Month 3	SFH intervention plus IAQ feedback vs. Usual services	-0·16 (-0·35 to 0·03)	0·10
	SFH intervention vs. Usual services	-0·09 (-0·30 to 0·11)	0·39
	SFH intervention vs. SFH intervention plus IAQ feedback	0·07 (-0·13 to 0·26)	0·50
Month 12	SFH intervention plus IAQ feedback vs. Usual services	0·03 (-0·14 to 0·19)	0·76
	SFH intervention vs. Usual services	0·07 (-0·11 to 0·25)	0·45
	SFH intervention vs. SFH intervention plus IAQ feedback	0·04 (-0·13 to 0·22)	0·62
**Post-hoc adjustment for prespecified confounders**			
Month 3	SFH intervention plus IAQ feedback vs. Usual services	-13·3 (-26·8 to 0·2)	0·05
	SFH intervention vs. Usual services	-10·4 (-25·0 to 4·2)	0·16
	SFH intervention vs. SFH intervention plus IAQ feedback	2·9 (-11·1 to 16·8)	0·69
Month 12	SFH intervention plus IAQ feedback vs. Usual services	-1·6 (-13·3 to 10·1)	0·79
	SFH intervention vs. Usual services	3·9 (-9·0 to 16·7)	0·55
	SFH intervention vs. SFH intervention plus IAQ feedback	5·5 (-6·6 to 17·6)	0·37
Log-transformed			
Month 3	SFH intervention plus IAQ feedback vs. Usual services	-0·14 (-0·32 to 0·04)	0·14
	SFH intervention vs. Usual services	-0·12 (-0·31 to 0·08)	0·25
	SFH intervention vs. SFH intervention plus IAQ feedback	0·02 (-0·17 to 0·21)	0·82
Month 12	SFH intervention plus IAQ feedback vs. Usual services	0·02 (-0·15 to 0·18)	0·85
	SFH intervention vs. Usual services	0·05 (-0·13 to 0·23)	0·57
	SFH intervention vs. SFH intervention plus IAQ feedback	0·04 (-0·14 to 0·21)	0·68

Table S21: Average $PM_{2·5}$ measurement (ug/m^3) by randomised group, time point and average baseline measurement (<35 / ≥35 ug/m^3)

Average $PM_{2·5}$ measurement (ug/m^3) - <35 at baseline		Usual services	SFH intervention	SFH intervention plus IAQ feedback	Total
Baseline (as randomised)	N	357	305	366	1028
	Mean (SD)	22·8 (6·7)	21·1 (6·5)	22·1 (6·3)	22·1 (6·5)
	Median (min, max)	23 (2, 34)	21 (8, 34)	22 (1, 34)	22 (1, 34)
Baseline (as analysed)	N	268	212	259	739
	Mean (SD)	22·9 (6·8)	20·9 (6·5)	22·0 (6·0)	22·0 (6·5)
	Median (min, max)	23 (2, 34)	20 (8, 34)	22 (5, 34)	22 (2, 34)
Month 3	N	268	212	259	739
	Mean (SD)	87·9 (84·3)	64·1 (63·6)	70·6 (70)	75·0 (74·5)
	Median (min, max)	53 (3, 403)	38 (6, 325)	38 (1, 345)	41 (1, 403)
Month 12	N	265	205	253	723
	Mean (SD)	59·5 (43·9)	63·2 (41·9)	58·8 (35·9)	60·3 (40·7)
	Median (min, max)	48 (11, 340)	54 (13, 265)	51 (14, 235)	51 (11, 340)
**Average $PM_{2·5}$ measurement (ug/m^3) - ≥35 at baseline**		**Usual services**	**SFH intervention**	**SFH intervention plus IAQ feedback**	**Total**
Baseline (as randomised)	N	215	237	266	718
	Mean (SD)	73·7 (47·5)	63·3 (30)	75·5 (53·3)	70·9 (45·3)
	Median (min, max)	57 (35, 251)	53 (35, 166)	59 (35, 422)	55 (35, 422)
Baseline (as analysed)	N	179	207	221	607
	Mean (SD)	76·2 (49)	63·7 (30·6)	75·5 (48)	71·7 (43·5)
	Median (min, max)	58 (35, 251)	53 (35, 166)	60 (35, 334)	57 (35, 334)
Month 3	N	179	207	221	607
	Mean (SD)	82·5 (90·8)	87·5 (99·1)	79·9 (84·2)	83·3 (91·4)
	Median (min, max)	36 (1, 417)	37 (6, 459)	37 (7, 353)	37 (1, 459)
Month 12	N	173	199	215	587
	Mean (SD)	72·8 (43·6)	74·8 (56)	74·0 (42·2)	73·9 (47·6)
	Median (min, max)	63 (11, 247)	62 (13, 389)	64 (14, 244)	63 (11, 389)

Supplementary Table S22: Intervention costs per household

	**Cost ($USD)**
**Intervention costs per household**	
Training cost for IAQ	2·8
Trainers	0·4
Trainees	1·2
Other related costs	1·2
Training cost for SFH	1·4
Trainers	0·3
Trainees	0·6
Other related costs	0·5
Delivery cost for IAQ	16·0
Dylos related costs	5·9
IAQ assessment	6·9
IAQ delivery	0·9
Other related costs	2·4
Delivery cost for SFH	1·5
Religious leaders	0·1
Other related costs	1·4

Supplementary Table S23: Sensitivity analysis - total costs, QALYs and ICER per household by trial groups

	No. of households	Costs			QALYs (SD)	ICER
		Intervention $USD	Healthcare $USD (SD)	Total $USD (SD)		
**Trial results**						
SFH intervention plus IAQ feedback	429	21·9	11·0 (22·0)	32·8 (22·0)	3·15 (1·11)	$980/QALY gained
SFH intervention	383	2·9	23·0 (61·0)	25·8 (61·0)	3.07 (1·10)	Dominated
Usual services	425	-	13·2 (30·4)	13·2 (30·4)	3·13 (1·16)	-
				**Incremental costs ($)**	**Incremental QALYs**	**ICER**
**Results of bootstrapped SUR models**						
SFH intervention plus IAQ feedback vs. usual services (mean, 95% CI)				19·6 (19·5 to 19·6)	-0·05 (-0·05 to 0·05)	Dominated
SFH intervention vs. usual services (mean, 95% CI)				12·1 (12·0 to 12·2)	-0·12 (-0·12 to -0·12)	Dominated

Supplementary Figures

Figure S1: Intervention logic model

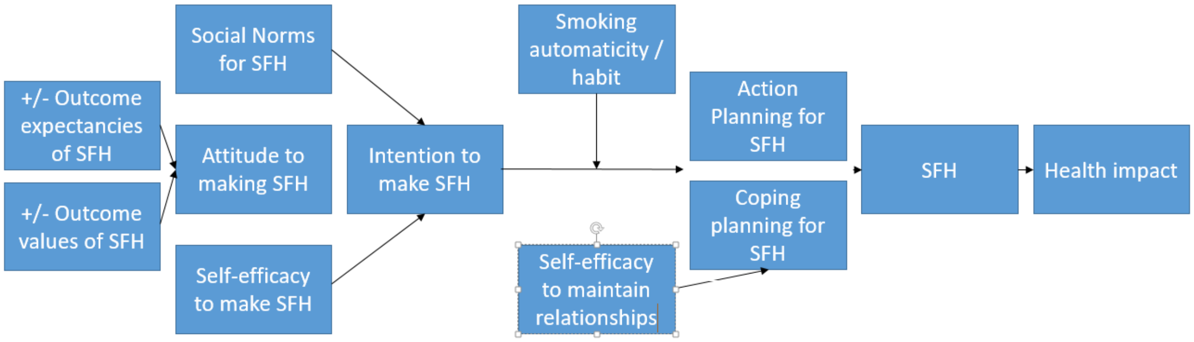

Figure S2: Untransformed outcome data - QQ-plot of the standardised residuals

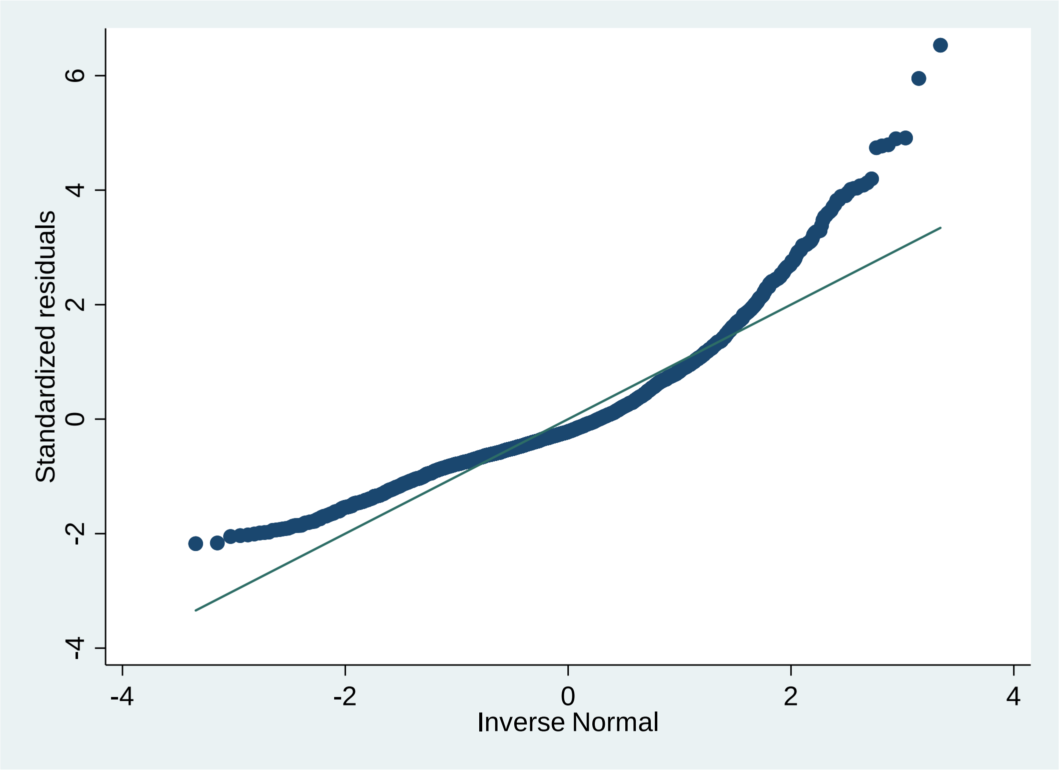

Figure S3: Untransformed outcome data - scatter plot of the standardised residuals against fitted values

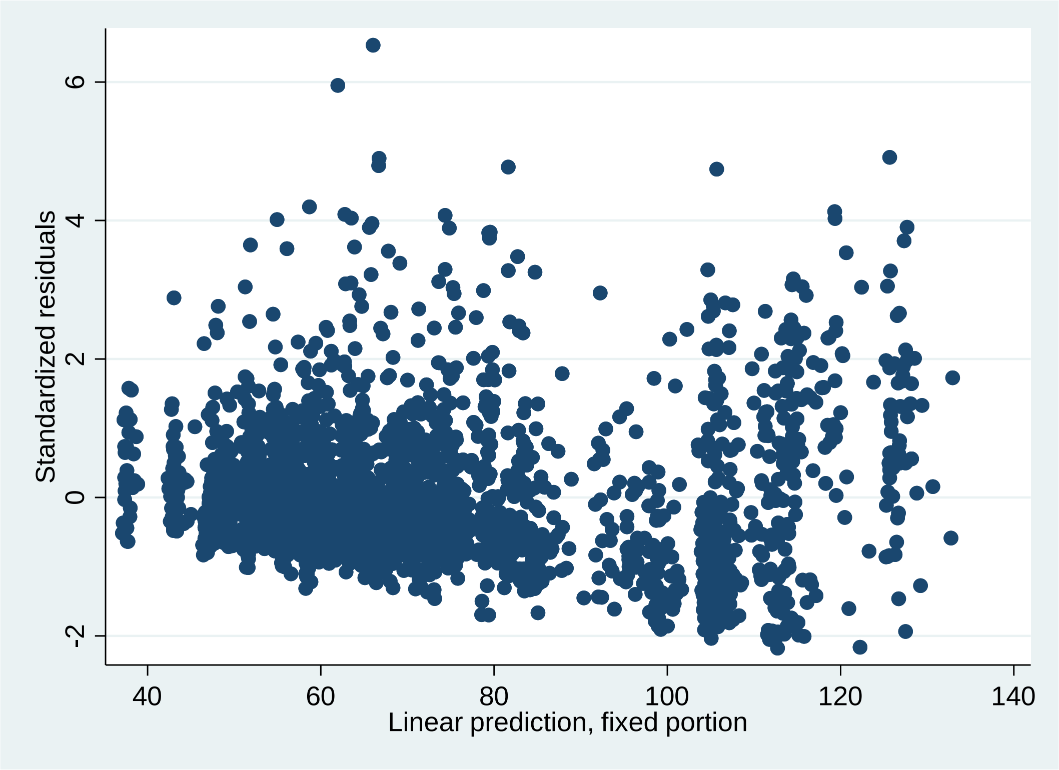

Figure S4: Log-transformed outcome data - QQ-plot of the standardised residuals

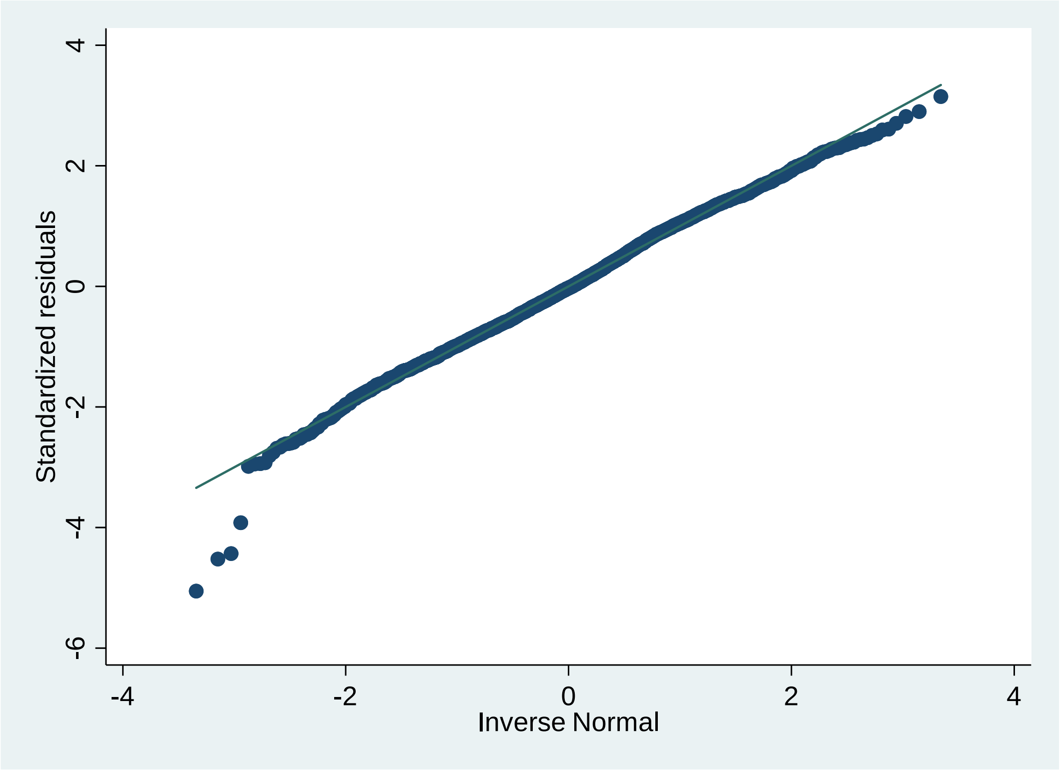

Figure S5: Log-transformed outcome data - scatter plot of the standardised residuals against fitted values

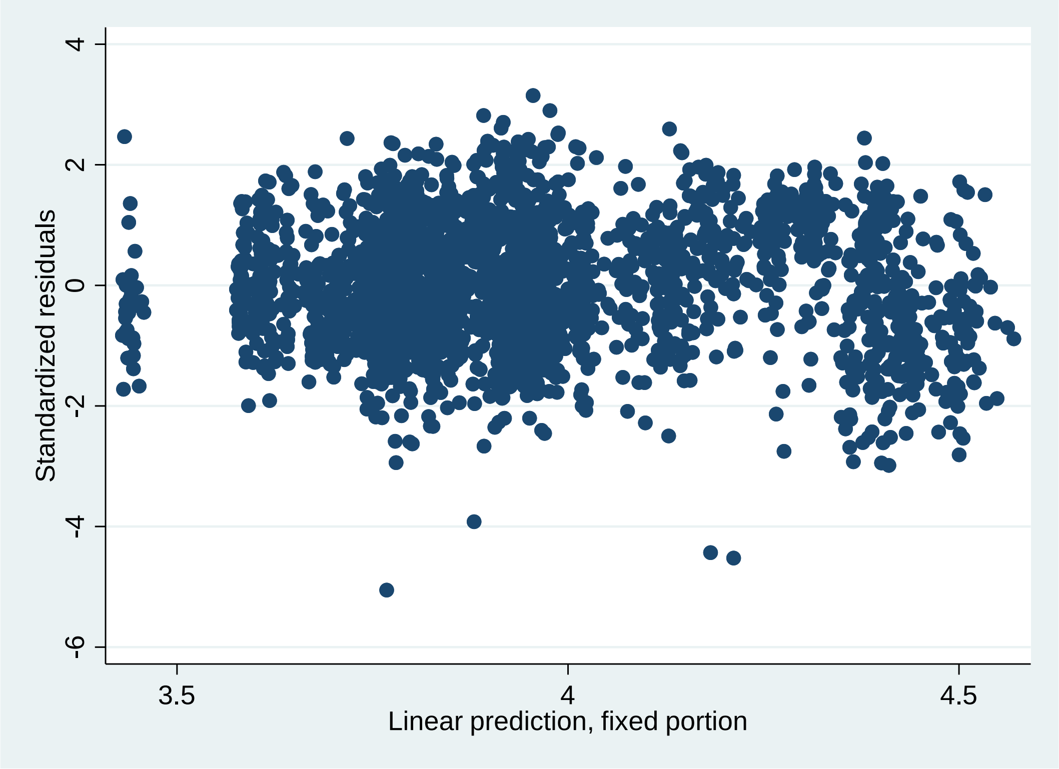

Figure S6: Predicted mean primary outcome results by trial group over time

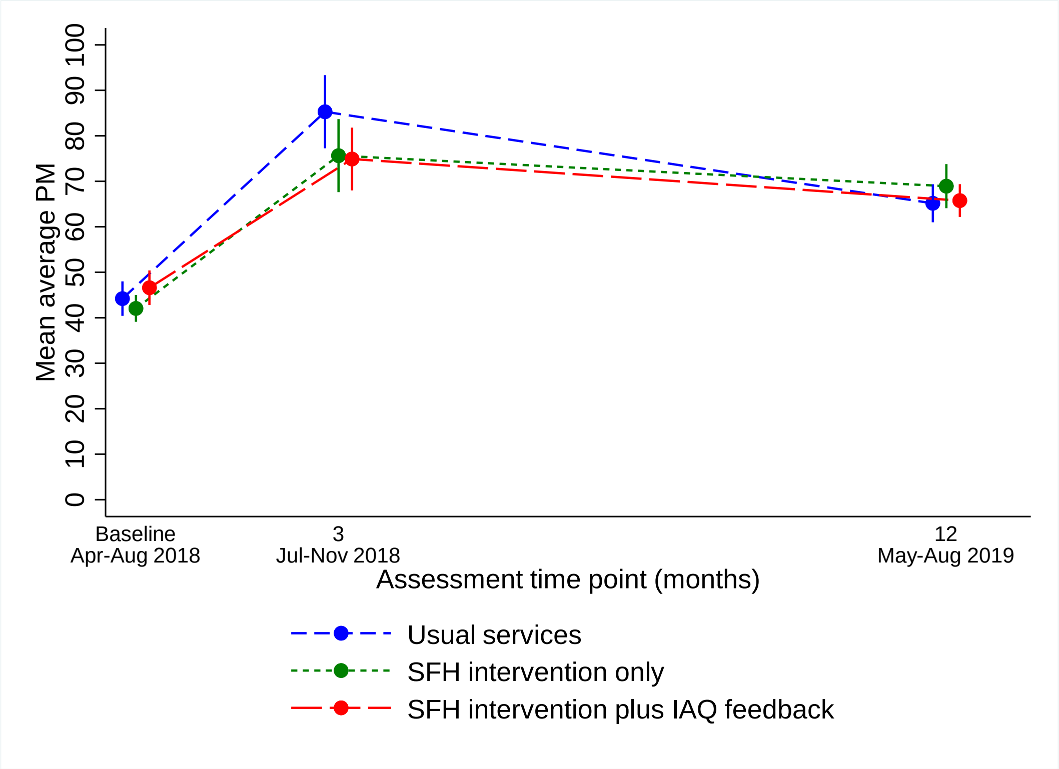

Figure S7: Sensitivity analysis - month three and 12 ‘uncorrected’

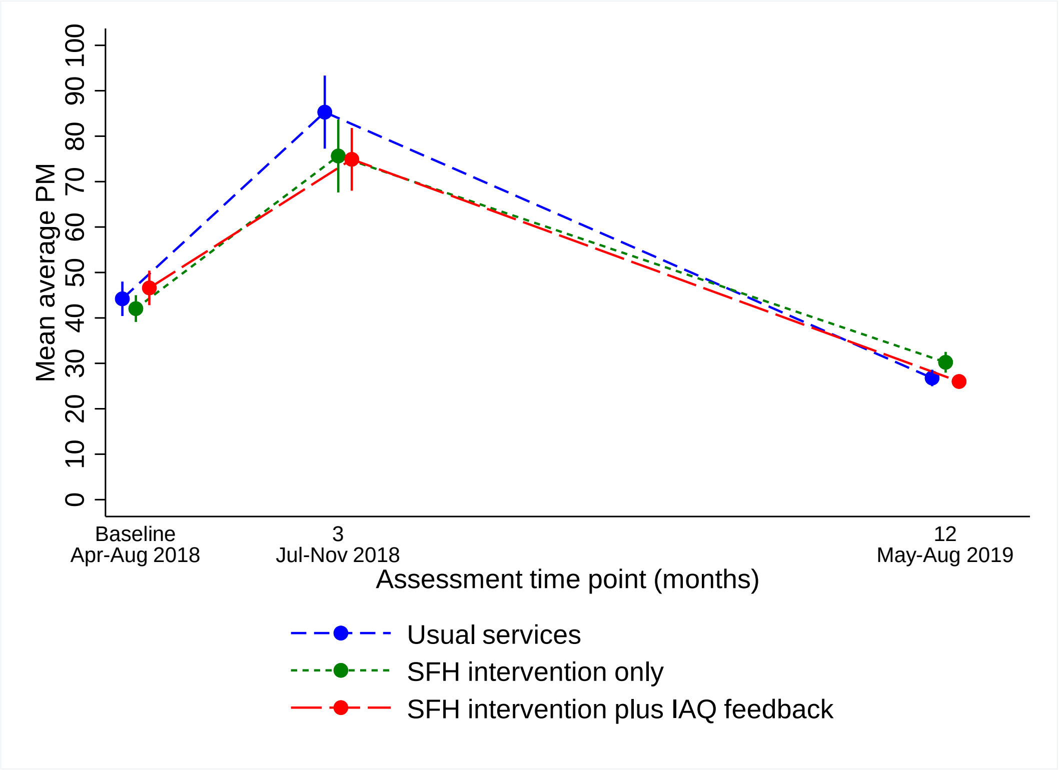

Figure S8: Sensitivity analysis - month three and 12 'corrected'

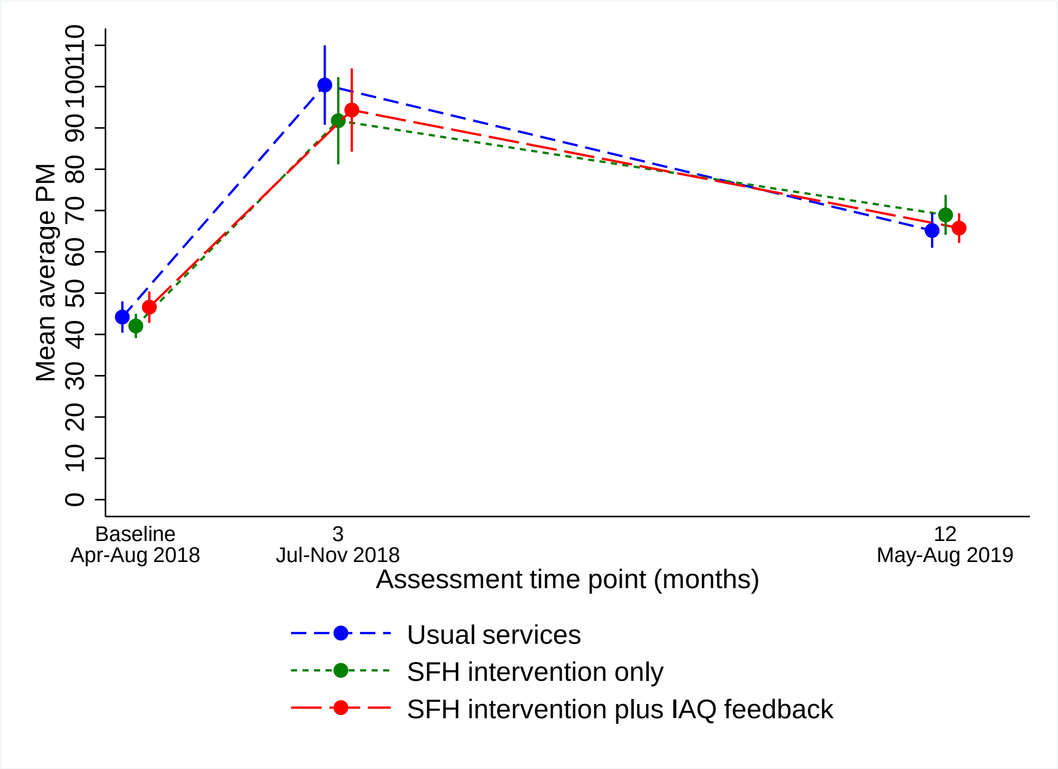

Figure S9: Dylos $PM_{2.5}$ measurements by randomised group over time for households with average PM <35 ug/m^3 at baseline

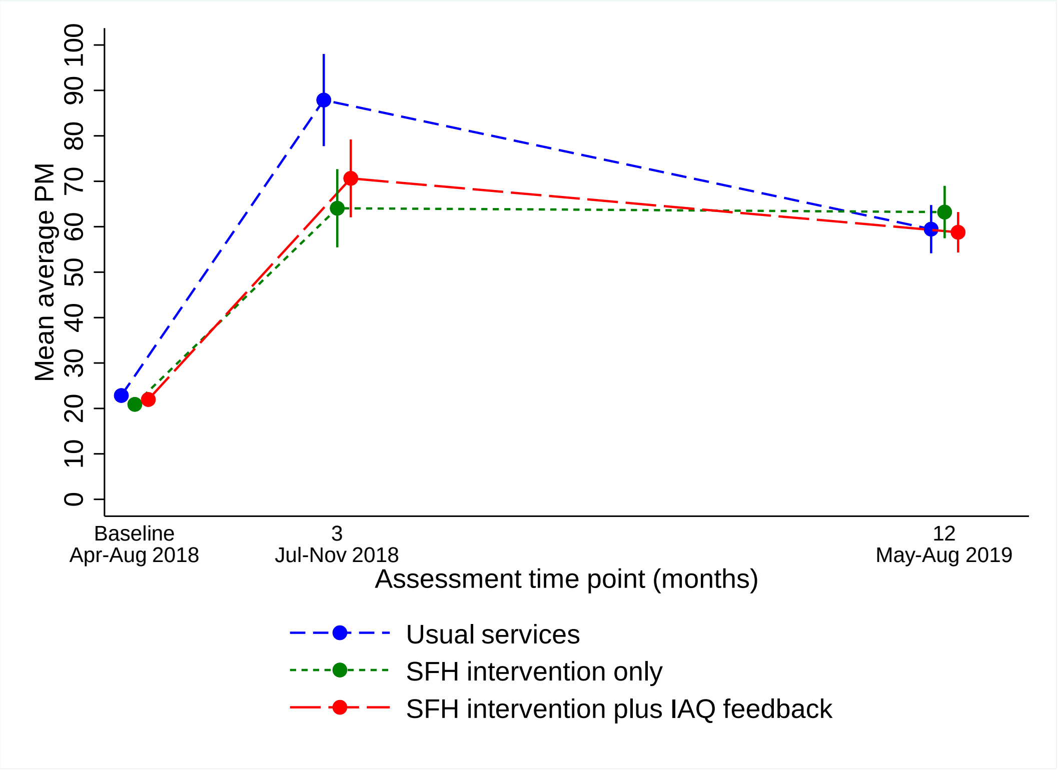

Figure S10: Dylos $PM_{2.5}$ measurements by randomised group over time for households with average PM ≥35 ug/m^3 at baseline

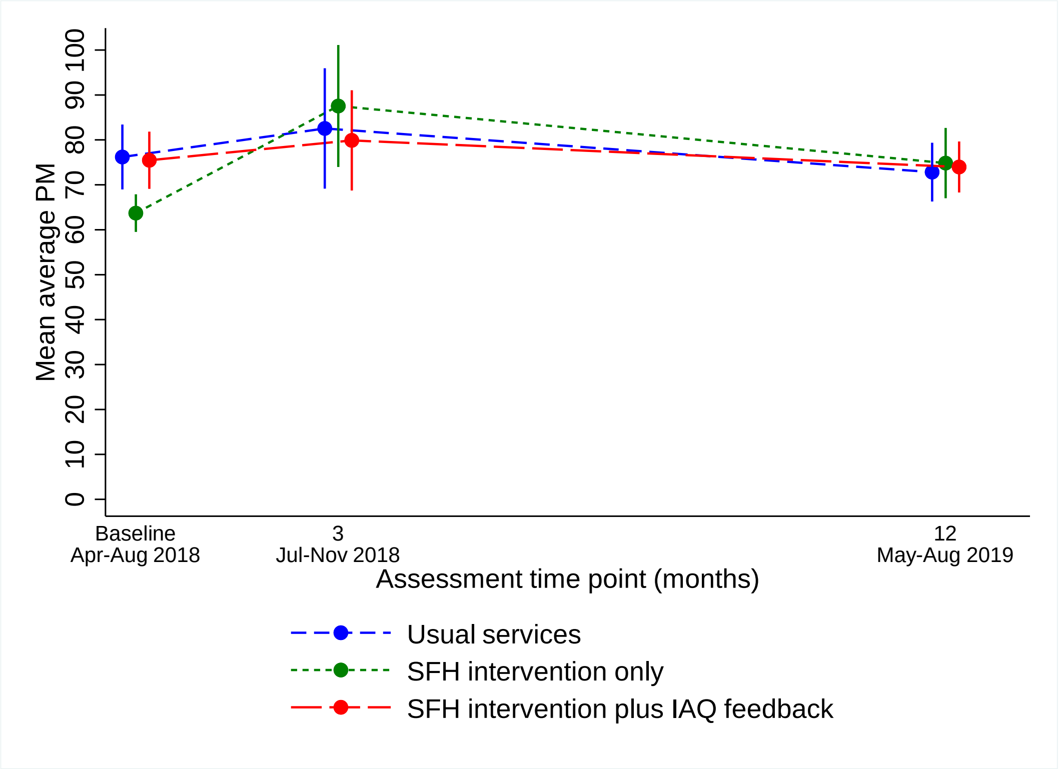

Figure S11: Cost-effectiveness plane of 5000 bootstrapped ICER replications

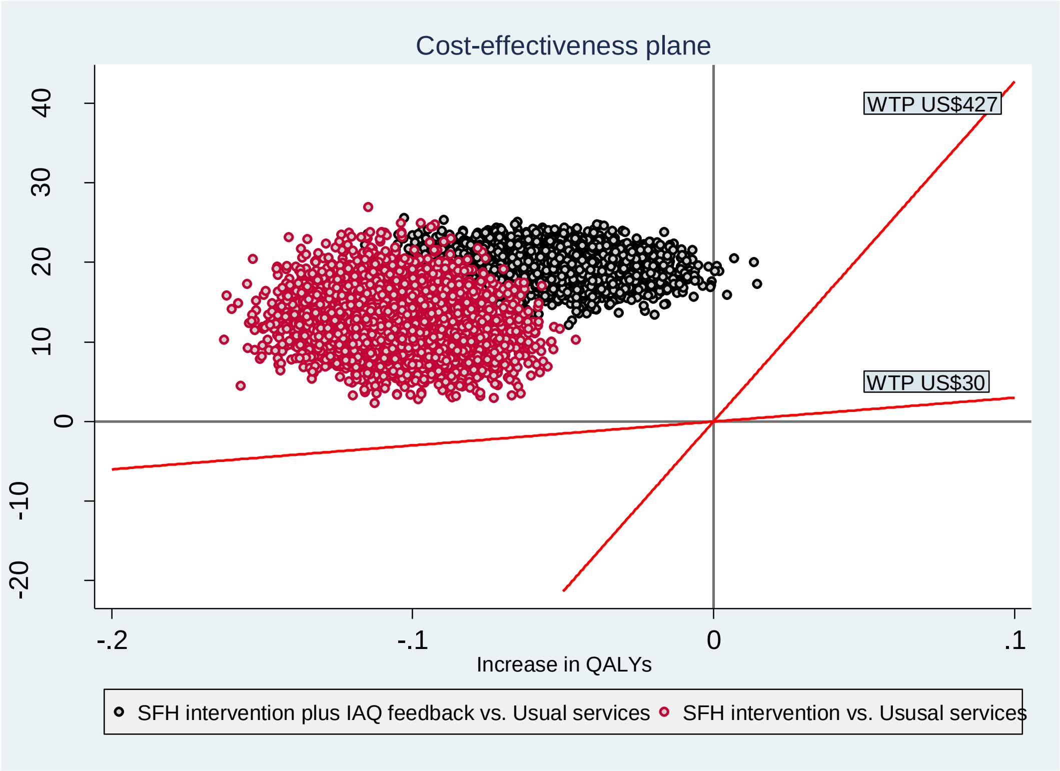

Figure S12: Sensitivity analysis - cost-effectiveness plane of 5000 bootstrapped ICER replications

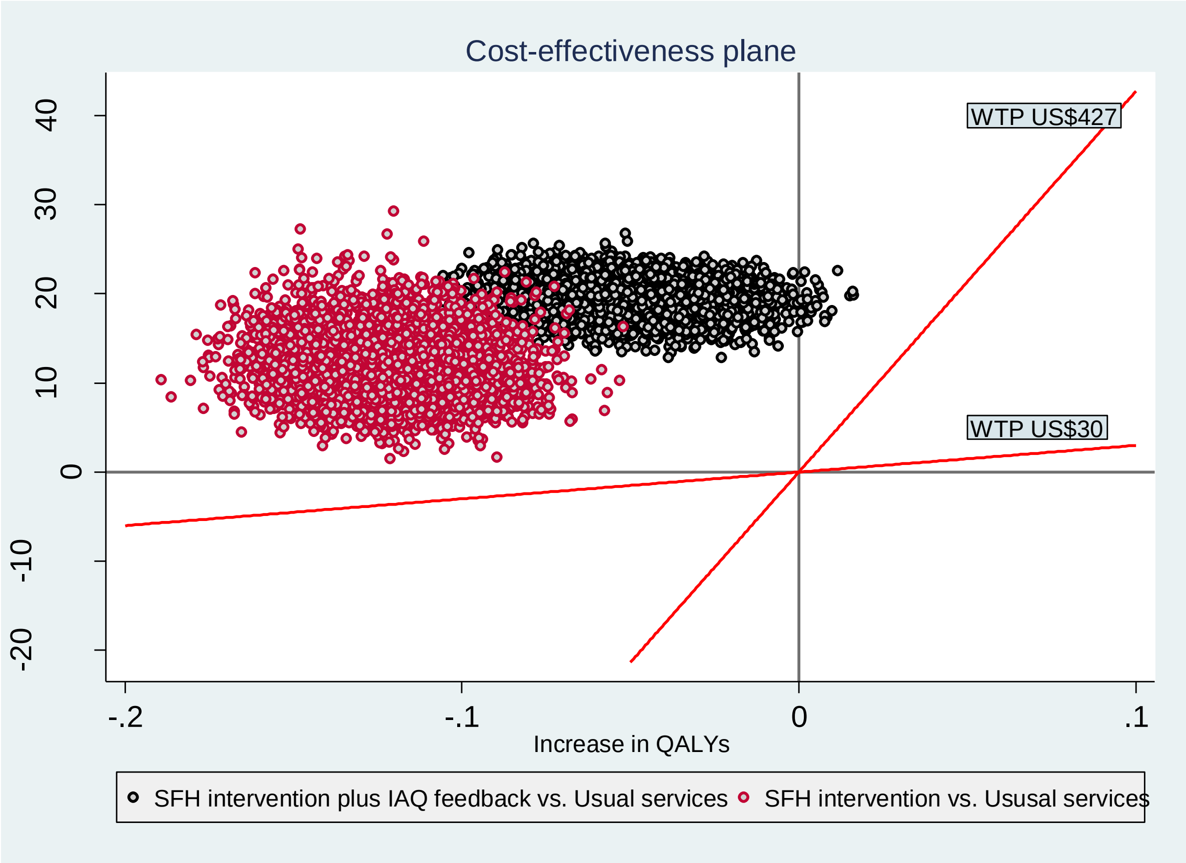

Supplement: Supplementary appendix 2 [file mmc2.pdf]
